# Simulated ocean acidification reveals winners and losers in coastal phytoplankton

**DOI:** 10.1371/journal.pone.0188198

**Published:** 2017-11-30

**Authors:** Lennart T. Bach, Santiago Alvarez-Fernandez, Thomas Hornick, Annegret Stuhr, Ulf Riebesell

**Affiliations:** 1 GEOMAR Helmholtz Centre for Ocean Research Kiel, Kiel, Germany; 2 Alfred-Wegener-Institut Helmholtz-Zentrum für Polar- und Meeresforschung, Biologische Anstalt Helgoland, Helgoland, Germany; 3 Leibniz Institute of Freshwater Ecology and Inland Fisheries (IGB), Experimental Limnology, Stechlin, Germany; University of Connecticut, UNITED STATES

## Abstract

The oceans absorb ~25% of the annual anthropogenic CO_2_ emissions. This causes a shift in the marine carbonate chemistry termed ocean acidification (OA). OA is expected to influence metabolic processes in phytoplankton species but it is unclear how the combination of individual physiological changes alters the structure of entire phytoplankton communities. To investigate this, we deployed ten pelagic mesocosms (volume ~50 m^3^) for 113 days at the west coast of Sweden and simulated OA (pCO_2_ = 760 μatm) in five of them while the other five served as controls (380 μatm). We found: (1) Bulk chlorophyll *a* concentration and 10 out of 16 investigated phytoplankton groups were significantly and mostly positively affected by elevated CO_2_ concentrations. However, CO_2_ effects on abundance or biomass were generally subtle and present only during certain succession stages. (2) Some of the CO_2_-affected phytoplankton groups seemed to respond directly to altered carbonate chemistry (e.g. diatoms) while others (e.g. *Synechococcus*) were more likely to be indirectly affected through CO_2_ sensitive competitors or grazers. (3) Picoeukaryotic phytoplankton (0.2–2 μm) showed the clearest and relatively strong positive CO_2_ responses during several succession stages. We attribute this not only to a CO_2_ fertilization of their photosynthetic apparatus but also to an increased nutrient competitiveness under acidified (i.e. low pH) conditions. The stimulating influence of high CO_2_/low pH on picoeukaryote abundance observed in this experiment is strikingly consistent with results from previous studies, suggesting that picoeukaryotes are among the winners in a future ocean.

## 1. Introduction

The seasonal succession of plankton involves the occurrence and disappearance of plankton taxonomic and functional groups in an annually repeated pattern [1]. The major biomass build-up during the spring bloom is traditionally seen as the starting point of the succession in temperate regions, although the initiation of the bloom already takes place in early winter [2,3]. Succession patterns differ among oceanographic regions and are controlled by a multitude of abiotic factors such as turbulence, nutrients, and light [4], as well as biotic interactions, including competition, predation, and infection [1,4,5].

Changes in the marine carbonate system due to the net influx of anthropogenic CO_2_ into the ocean’s surface layer (i.e. ocean acidification (OA)) could alter phytoplankton succession because taxonomic groups shaping the succession pattern are differently sensitive to changing carbonate chemistry. Phytoplankton species which benefit from CO_2_ fertilization may become more dominant in future communities while those which are unresponsive to increasing CO_2_ or even detrimentally affected by decreasing pH could become less important or be replaced by other species [6–9]. Uncovering the potential for CO_2_-induced community shifts is important as these can re-organize the energy flow through food webs and alter biogeochemical element fluxes [10,11].

In this study we investigated the influence of projected end-of-the century carbonate chemistry conditions (average pCO_2_ = 760 μatm) on a natural winter-to-summer phytoplankton succession in a temperate coastal environment. Our study is part of the BIOACID II long-term mesocosm study which took place in Gullmar Fjord (Skagerrak, west coast of Sweden) from January to July 2013. It belongs to a series of papers covering various components of the plankton community in and outside the mesocosms. A summary on the main foci of these contributions is provided in the overview paper accompanying this PLOS collection [12]. The focus in the present contribution is primarily on how CO_2_ affects phytoplankton functional and taxonomic groups during the winter-to-summer succession.

## 2. Methods

### 2.1 Mesocosm deployment, manipulation, and maintenance

On the 29^th^ of January 2013, ten “Kiel Off-Shore Mesocosms for Future Ocean Simulations” (KOSMOS, M1-M10; [13]) were moored by research vessel *Alkor* in Gullmar Fjord on the Swedish west coast (58° 15’ 50” N, 11° 28’ 46” E). Study site, key events, deployment, and mesocosm manipulation procedures are described in detail in the abovementioned overview paper [12]. In brief, each mesocosm was composed of an 8 m tall floatation frame and an 18.7 m long cylindrical polyurethane bag with a diameter of 2 m. The bags were folded and installed in the floatation frame before mesocosm deployment by *Alkor*. After deployment, bags were unfolded and lowered underwater to allow water exchange with the fjord. Water inside the bags was isolated from the fjord water by attaching 2 m long conical sediment traps to the bottom and pulling the upper end of the bag about 1.5 m above the surface [13,14]. The mesocosm bags with the attached sediment traps reached ~19 m deep after the closing procedure.

Extended sea ice coverage prolonged the time between mesocosm deployment and the closing procedure. They were closed for the first time on the 12^th^ of February and CO_2_ was manipulated in the high CO_2_ mesocosms (M2, M4, M6, M7, M8) shortly thereafter. However, due to technical difficulties with the sediment traps we had to stop the experiment after 19 days on the 3^rd^ of March. Afterwards, mesocosms were lowered below surface to allow water exchange while the sediment traps were repaired on land. After four days we restarted the experiment by closing the mesocosms for the second time on the 7^th^ of March. The second experiment lasted for 113 days until the 28^th^ of June. In the present paper we only describe results from the second experiment. We will use the “experimental day nomenclature” which is consistent among all papers associated with this mesocosm study. Here, the 7^th^ of March is day -2 and the 28^th^ of June is day 111.

The mesocosms enclosed a volume ranging from 47.5 (M3) to 55.9 (M2) m^3^ [12]. The water was gently mixed directly after enclosure by bubbling the water column for 5 minutes with compressed air. A second bubbling procedure two days after enclosure (day 0) was necessary to fully eliminate the salinity stratification. All mesocosms were cleaned by divers from the outside approximately every second week and from the inside with a cleaning ring approximately every 8^th^ day. A mesh (1 mm mesh size) was attached to the cleaning ring on day 6 to remove large zooplankton (e.g. jelly fish) or nekton (e.g. fish) from the water column as these organisms were considered to be unevenly distributed among mesocosms. However, only very few organisms, mostly jelly fish and some fish larvae, were removed during this operation.

Five of the ten mesocosms were enriched with CO_2_-aerated seawater at the beginning of the experiment (M2, M4, M6, M7, M8) while the other five mesocosms remained unperturbed and served as controls (M1, M3, M5, M9, M10). High CO_2_ concentrations had to be re-established on 5 occasions (days 17, 46, 48, 68, 88) during the study to compensate for CO_2_ gas loss at the air-sea boundary layer of the mesocosms.

Due to the long duration of the experiment, we added 22 L of unfiltered fjord water to each mesocosm on every 4^th^ day thereby allowing plankton species which were not present during closing to enter the mesocosm [12]. Green sea-urchin (*Strongylocentrotus droebachiensis*) and herring (*Clupea harengus*) larvae were added to each mesocosm on day 56 and day 63, respectively, to study the influence of OA on their development. Both species were added in relatively low densities (~90 herring eggs and 110 sea urchin larvae per m^3^) to minimize potential top-down-effects [12]. Please note that the OA response of these particular organisms will be addressed in detail in other publications.

Ethical statement: Herring welfare was assured by performing the experiment according to the ethical permission (number 332–2012) issued by the Swedish Board of Agriculture "Jordbruksverket"). The species (*Clupea harengus*) used is not endangered and was obtained from a local registered and licensed fisherman (licence ID = 977 224 357).

### 2.2 Sampling, filtration, and measurements

All mesocosms were sampled every second day for usually 1–3 hours starting at 9 a.m. (local time) from small boats. The water column was sampled with integrating water samplers equipped with pressure sensors (IWS, Hydrobios) which collect 5 L of seawater evenly from the water column while being lowered from 0–17 m. Water from two IWS hauls were transferred with a tube into a 10 L carboy. The carboys were brought back to shore and stored in a temperature-controlled room set to *in situ* water temperature. Subsamples for particulate matter (PM), flow cytometry, light microscopy, and pigment analysis were taken from carboys shortly (usually within 1 hour) after they arrived in the temperature-controlled room. Each carboy was rotated gently before subsampling in order to avoid sedimentation bias.

PM samples were filtered (500 mL, Δpressure -200 mbar) on glass fiber (GF/F) filters and immediately photographed at full magnification with a CANON 60D and a EF-S 60 mm f/2.8 Macro lens. These pictures were manually processed to count the abundance of the large (>200 μm) diatom *Coscinodiscus concinnus*.

Pigment samples were filtered (800 mL, Δpressure -200 mbar) on GF/F filters. These filters were folded, put into 2 mL cryovials, and stored at -80°C for 4–7 months until analysis. Pigments were extracted in 90% acetone and their concentrations quantified by means of reverse phase high performance liquid chromatography (HPLC) [15]. The contribution of distinct phytoplankton taxa to total chlorophyll *a* (chl*a*) was calculated with the CHEMTAX software which classifies phytoplankton based on taxon-specific pigment ratios [16]. For the calculation, we used pigment ratios provided by Mackey et al. [16]. Pigment data from all mesocosms were aggregated in one data sheet and evaluated in the same analysis run (iterations = 86, root-mean-square error = 0.22). Thus, the in- and output pigment ratios used in the CHEMTAX analysis were identical in all mesocosms (S1 Table).

Light microscopy samples were transferred from the carboys into 250 mL brown glass bottles and fixed with acidic Lugol’s solution (1% final concentration). Phytoplankton were counted and identified 1–24 months after sampling at 200 and 400 times magnification with an Zeiss Axiovert inverted microscope [17]. Light microscopic species counts comprised auto-, mixo-, and heterotrophic protists within a size range from ~10–500 μm. A species list with approximate organism sizes is provided in S2 Table.

Cells smaller than 10 μm were abundant in the experiment but could often not be taxonomically identified. The exceptions were small silicifying species (<5 μm) which were identified with scanning electron microscopy. Therefore, ~10 mL of water sample were gravity filtered on 0.2 μm polycarbonate filters and further processed as described by Bach et al. [18].

Flow cytometry subsamples for phytoplankton analyses were transferred from the 10 L carboys into 50 mL beakers directly after return of the sampling boats in the harbor. Subsamples (650 μL per mesocosm) were immediately analyzed within 3 hours using a Accuri C6 (BD Biosciences) flow cytometer [12]. The flow rate estimated by the Accuri C6 was controlled and verified regularly by weighing sample before and after measurements and calculating the volume difference. Phytoplankton populations were distinguished based on the signal strength of the forward light scatter (FSC), the red fluorescence from chlorophyll *a* light emission (FL3), and the orange fluorescence from phycoerythrin light emission (FL2) [19]. The FSC signal strength is positively correlated with particle size and can be used to distinguish phytoplankton size classes [20]. To constrain the size range we conducted sequential fractionations using polycarbonate filters of different pore sizes (0.2, 0.8, 2, 3, 5, 8 μm) and gravity filtration [21]. The following groups were defined (S1 Fig): *Synechococcus* (0.8–3 μm), Cryptophytes (Crypto, 2–8 μm), Picoeukaryotes (Peuks, 0.8–3 μm), and four groups of Nanoeukaryotes with increasing size (Nano I–III between 2–8 μm with Nano III being the largest; Nano IV, >8 μm). The clusters of Peuk and Nano I–IV populations changed their appearance slightly in the course of the study. We accounted for these changes by adjusting gate shapes in the cytograms on 7 occasions (days 14, 30, 46, 66, 79, 94, 108). Importantly, gates and gate adjustments were identical in all mesocosms at every point in time. This was essential to keep the flow cytometry data comparable among replicates and treatments. The abundance of each flow cytometry group (in cells mL^-1^) was calculated as the number of events within a gate divided by the analyzed volume.

Bacterial abundance samples were withdrawn from the 10 L carboys directly after sampling and immediately fixed with glutaraldehyde (0.5% v/v; 30 minutes), flash-frozen in liquid nitrogen, and stored at -80°C for 4–7 months until measurements with the Accuri C6 flow cytometer. Bacteria samples were prepared for analysis by thawing samples at 37°C and staining them with SYBR green I for 15 minutes at 20°C in the dark. Bacteria could be distinguished from non-living particles by the green fluorescence of the stained DNA and from phytoplankton by the lack of red fluorescence [22]. Please note that archaea are also included in this analysis but they are typically much less abundant in surface waters than bacteria and therefore not specifically considered here as a separate group [23].

### 2.3 Statistical data analysis

**Fig 1:**
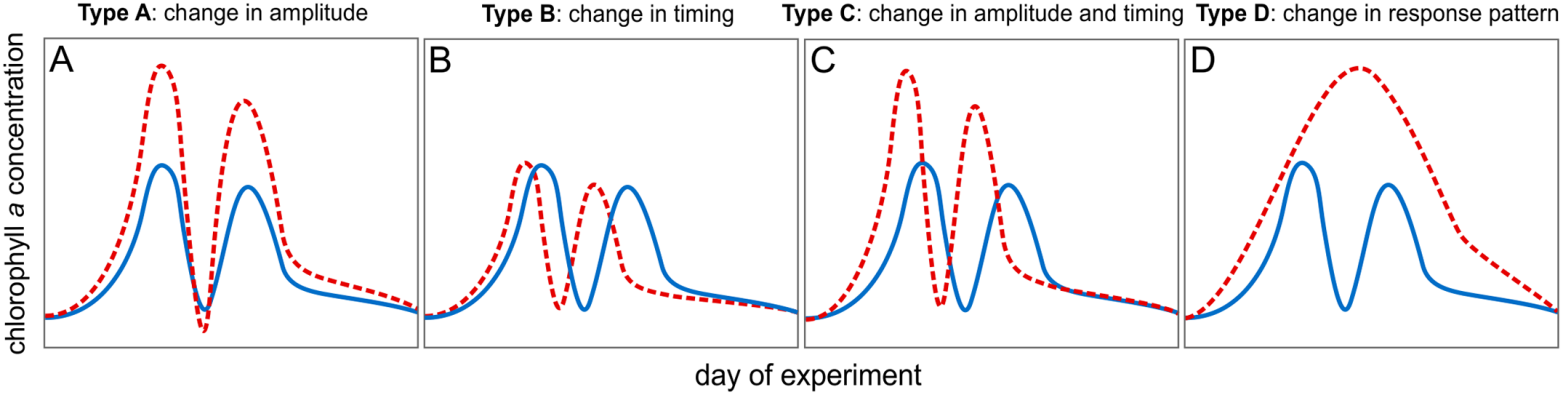
Four potential scenarios how phytoplankton bloom development could be altered by ocean acidification explained with the example of chl*a* concentration. Blue and red lines illustrate control and “treatment”, respectively. (A) Change in bloom amplitude. (B) Change in bloom timing. (C) Change in bloom amplitude and timing. (D) Change in bloom pattern.

We postulated that the high CO_2_ treatment can affect the temporal trend of a dependent variable in four different ways: by amplification or weakening of the peak amplitudes (Fig 1A); by inducing a shift in peak timing (Fig 1B); by a changing peak amplitude and timing (Fig 1C); by changing the entire pattern of the response curve (Fig 1D). To detect and visualize such potential responses we applied generalized additive mixed-effect modelling (GAMM; R packages “mgcv” and “nlme” [24–26]) and analyzed the data with the following procedure. Three different GAMM models were fitted to each dependent variable as a function of time (in our case as a function of the day of experiment; e.g. chl*a*(day of experiment)). CO_2_ was set as categorical explanatory variable modifying the absolute values of the dependent variable and/or its trend shape over time [25,27]. The first model assumed no difference between treatments. The second model assumed a more or less constant offset in temporal trends but no change in trend shape (i.e. phenology). The third model assumed differences in phenology. In each model, the mesocosm number was set as random effect to account for any unknown effects of individual mesocosms. We accounted for heteroscedasticity and temporal autocorrelation of residuals in the models to ensure that model assumptions were satisfied [26]. In most cases best fitting results were gained with a 3^rd^ order autoregressive structure and a variance configuration accounting for within-treatment variance. Statistically significant GAMM models were compared by their coefficient of correlation (R^2^). The model with the highest R^2^ was chosen as the one describing the response best. With this approach, CO_2_ effects were detected by determination of the most appropriate GAMM model (model 1 = no CO_2_ effect; model 2,3 = CO_2_ effect detected). Importantly, model 2 was in no case gaining the highest R^2^ value. Thus, when a CO_2_ effect was detected, it was always an effect on phenology (model 3). The response scenario (Fig 1) was determined in case of a detected CO_2_ effect by visually inspecting the phenology of the GAMM model fits shown in S2 Fig.

## 3. Results

### 3.1 Basic chemical parameters and the different phases of phytoplankton blooms in control and high CO_2_ mesocosms

The partial pressure of CO_2_ was elevated to ~1000 μatm in the high CO_2_ mesocosms during the first six days of the study. CO_2_-outgassing at the air-sea interface in the high CO_2_ mesocosms was countered by regular additions of CO_2_-aerated water while pCO_2_ was not manipulated in the control treatment. The pCO_2_ levels averaged over the entire experimental period were 759 (±11) and 384 (±19) μatm in the high CO_2_ and control environments, respectively [12].

**Fig 2:**
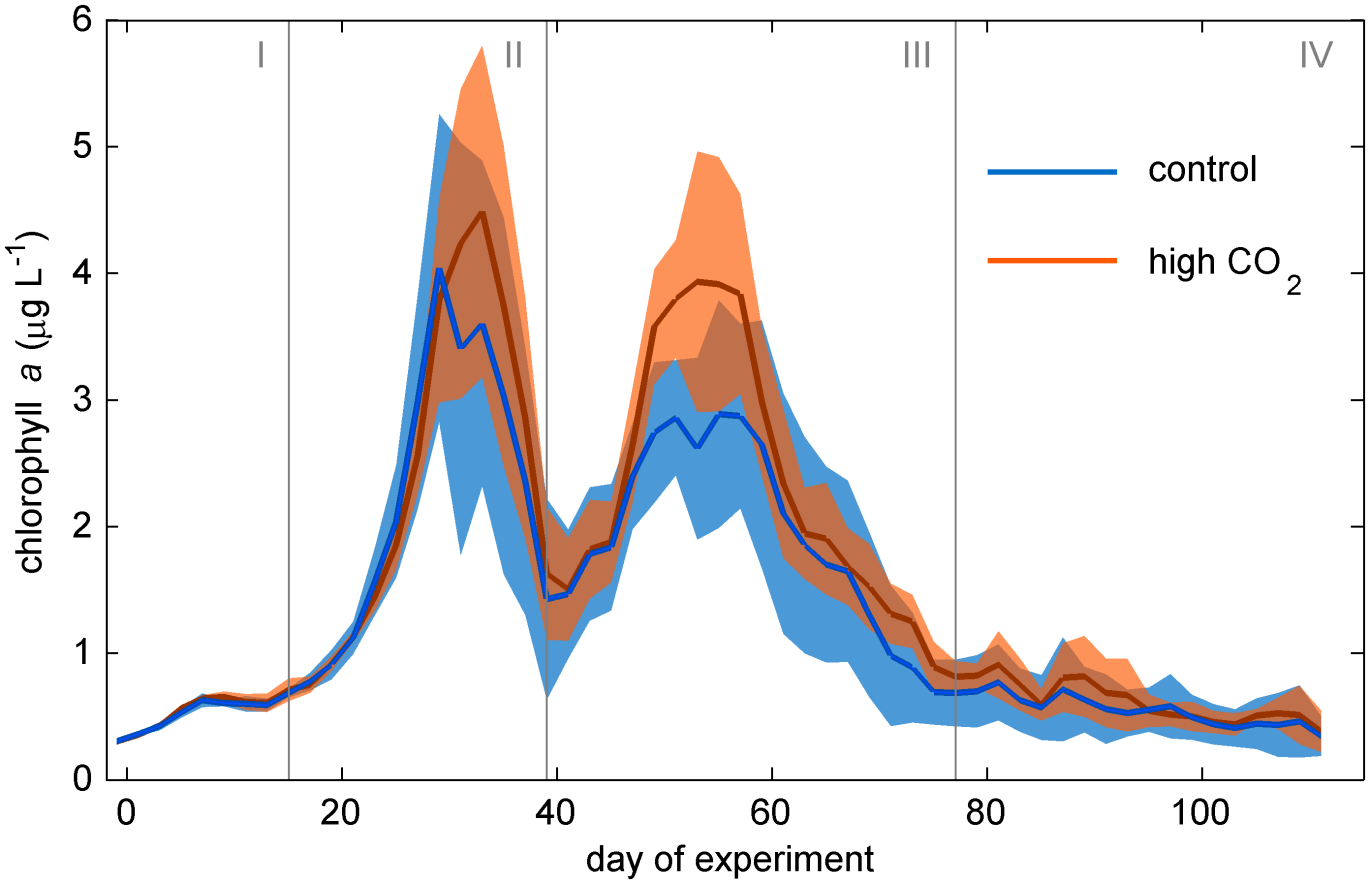
Chl*a* development over time. Red and blue lines display the average of five high and five ambient CO_2_ mesocosms, respectively. Shaded areas represent standard deviations from means. Vertical grey lines (Roman numbers I to IV) separate the four experimental phases.

Inorganic nutrients were up-welled by winter mixing before the study started and enclosed with comparable concentrations in all mesocosm bags when isolating the water from the surrounding fjord (S3 Table; see also [12]). The subsequent temporal development of nutrient concentrations was similar in control and high CO_2_ mesocosms. NO_3_^-^+NO_2_^-^ concentrations dropped to values close to detection limit at the first chl*a* peak (around day 33; Fig 2) and remained at these low values until the end of the experiment (0.04 ±0.01 μmol kg^-1^). PO_4_^3-^ and Si(OH)_4_ concentrations were also quite low at the first chl*a* peak (0.07 ±0.07 and 2.67 ± 0.30 μmol kg^-1^ on day 33, respectively) but those nutrients were not the primarily limiting ones after the spring bloom [12]. Si(OH)_4_ remained above detection limit for some days to weeks after day 33 before reaching detection limit. PO_4_^3-^ fluctuated at a very low level (max. 0.2 μmol kg^-1^) from day 33 until the end of the experiment [12]. The average inorganic nutrient concentrations (NO_3_^-^+NO_2_^-^, PO_4_^3-^, Si(OH)_4_, and NH_4_^+^) for each of the four phases are provided in S3 Table. A graphical representation of the inorganic nutrient dataset is provided in the overview paper [12] accompanying this study.

The temporal development of Chl*a* was similar in control and high CO_2_ mesocosms (Fig 2). Chl*a* concentrations were initially low (~0.3 μg L^-1^) and showed a relatively slow increase until day 17. Afterwards, chl*a* started to increase rapidly until reaching the first of two major peaks around day 33 (Fig 2). The first bloom declined after day ~33 with chl*a* concentrations dropping to ~1.5 μg L^-1^ on day ~40. This temporal minimum of chl*a* also marks the initiation of the second phytoplankton bloom which peaked around day 55 (Fig 2). Peak chl*a* concentrations were on average only slightly lower than in the first bloom with peak1/peak2 chl*a* ratios ranging from 1.5 (M10) to 0.9 (M3) [12]. The second chl*a* peak declined more slowly and reached baseline values around day 77 (Fig 2). After day 77, chl*a* concentrations remained at low concentrations (~0.5 μg L^-1^) and no further chl*a* peak developed (Fig 2).

Based on the observed chl*a* development we divided the experiment in 4 major phases (Fig 2). Phase I is the time before the first major chl*a* build-up and characterized by relatively low chl*a* (day -2–16). Phase II comprises the build-up and decline of the first major chl*a* peak (day 17–40). Phase III includes the second major chl*a* peak (day 41–77). Phase IV is the post-bloom period where chl*a* was relatively low and fairly stable (day 78–111).

### 3.2 Succession of functional and taxonomic phytoplankton groups in control and high CO_2_ mesocosms

The plankton community composition was similar among mesocosms at the beginning of the study (see [12] for a detailed analysis). Likewise, the succession of phytoplankton groups was similar in all mesocosms so that the following description of the temporal development refers to both the control and the high CO_2_ treatment.

**Fig 3:**
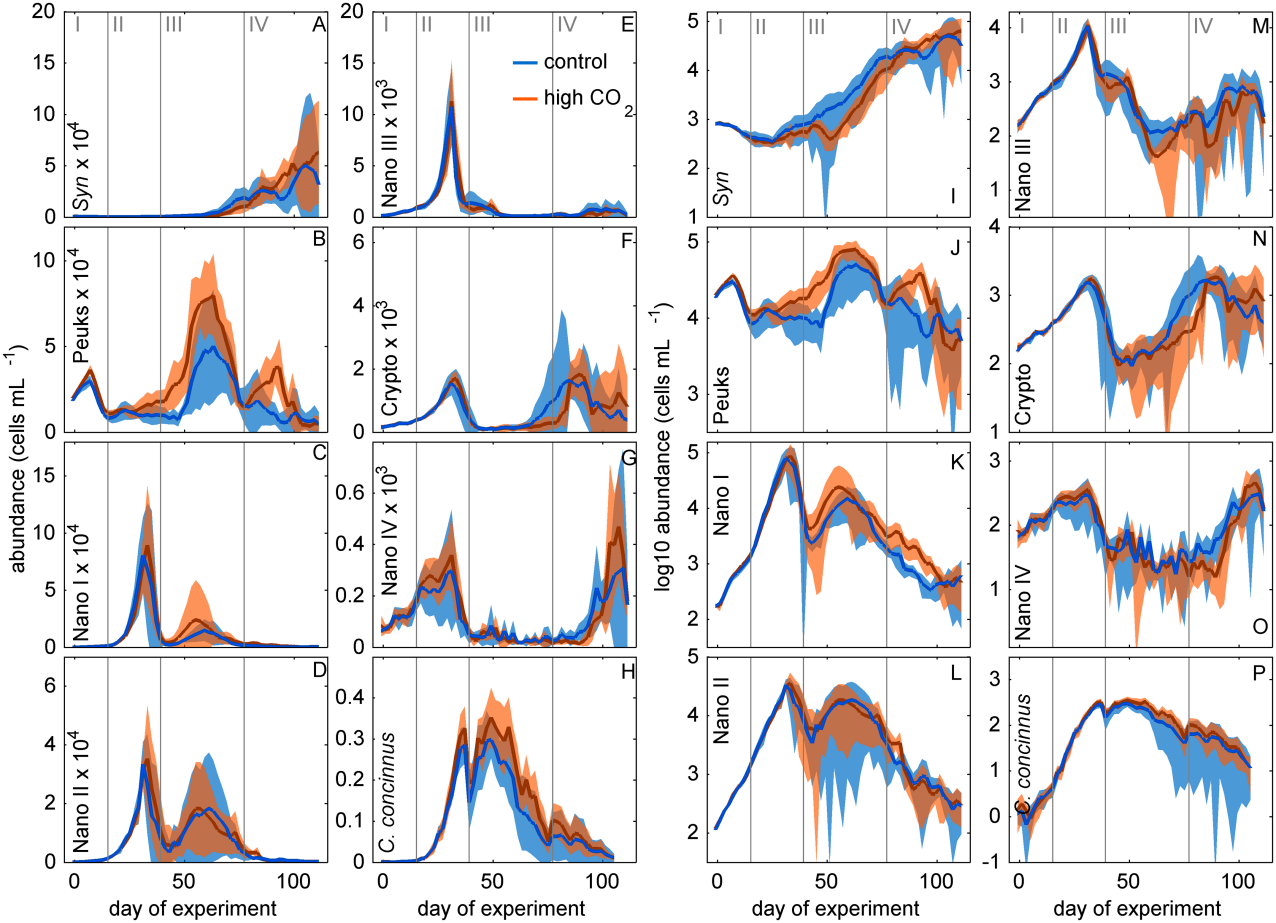
Development of phytoplankton groups quantified by flow cytometry and filter counts. Red and blue lines display the average of five high and five ambient CO_2_ mesocosms, respectively. Shaded areas represent standard deviations from means. Data are displayed on linear (A-H) and logarithmic y-axis (I-P). Note: the exponent in A-H after a group name needs to be multiplied with the y-axis numbering (e.g. 5 Syn x 10^4^ → 50000 *Synechoccocus* cells mL^-1^). Vertical grey lines (Roman numbers I to IV) separate the four experimental phases.

**Fig 4:**
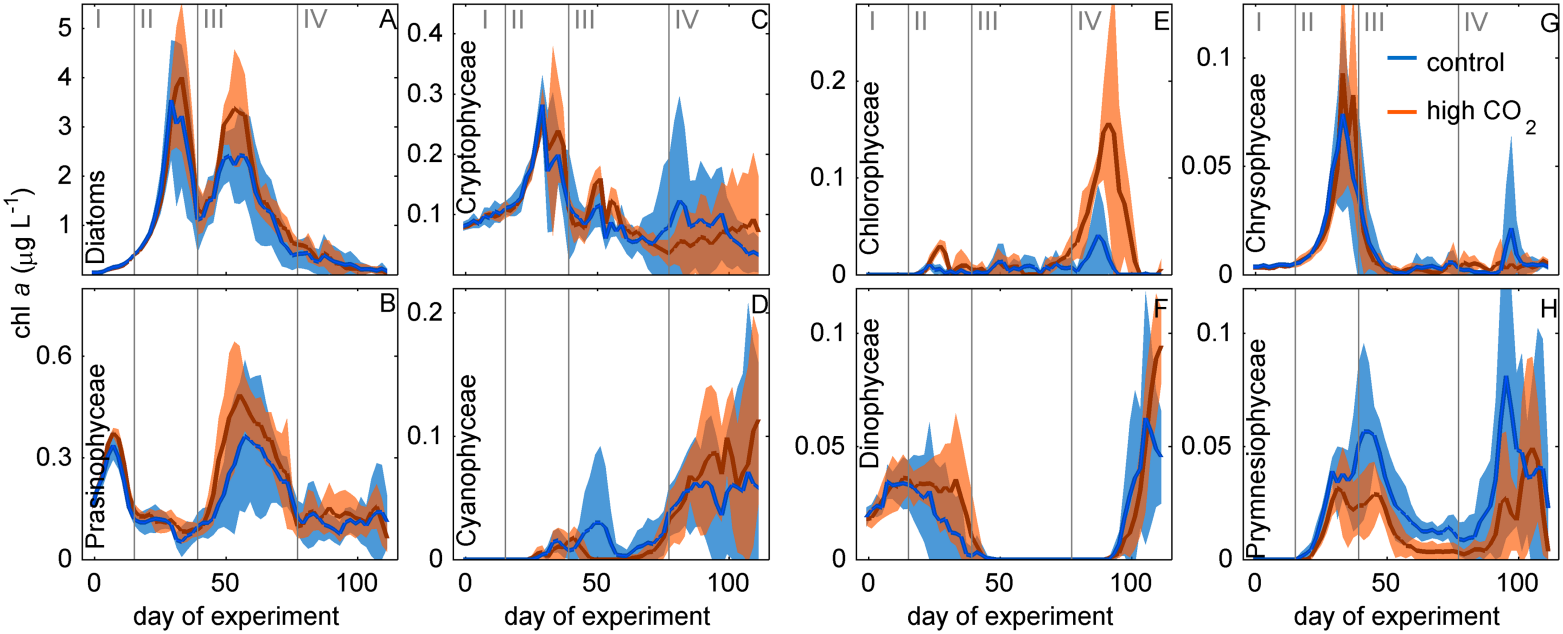
Development of phytoplankton classes based on CHEMTAX pigment taxonomy. Red and blue lines show the average of five high and five ambient CO_2_ mesocosms, respectively. Shaded areas represent standard deviations from means. The y-axis shows the amount of chl*a* contributed by each class. Vertical grey lines (Roman numbers I to IV) separate the four experimental phases.

**Fig 5:**
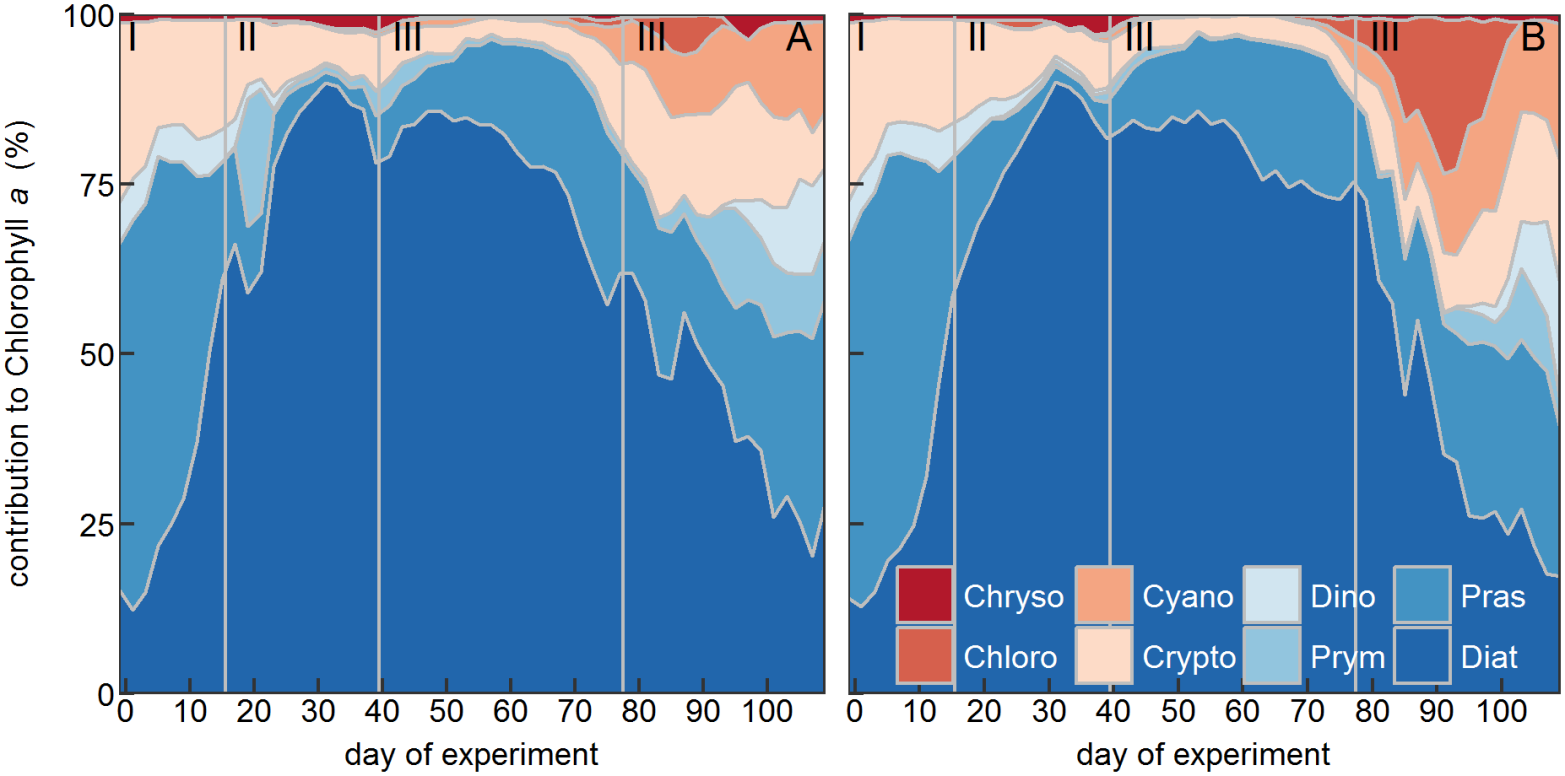
Relative chl*a* contribution of the 8 phytoplankton classes determined with CHEMTAX to bulk chl*a*. (A) Average of the control mesocosms. (B) Average of the high CO_2_ mesocosms. Vertical grey lines (Roman numbers I to IV) separate the four experimental phases. Chryso = Chrysophyceae; Cyano = Cyanophyceae; Dino = Dinophyceae; Pras = Prasinophyceae; Chloro = Chlorophyceae; Crypto = Cryptophyceae; Prym = Prymnesiophyceae; Dia = Diatoms.

**Fig 6:**
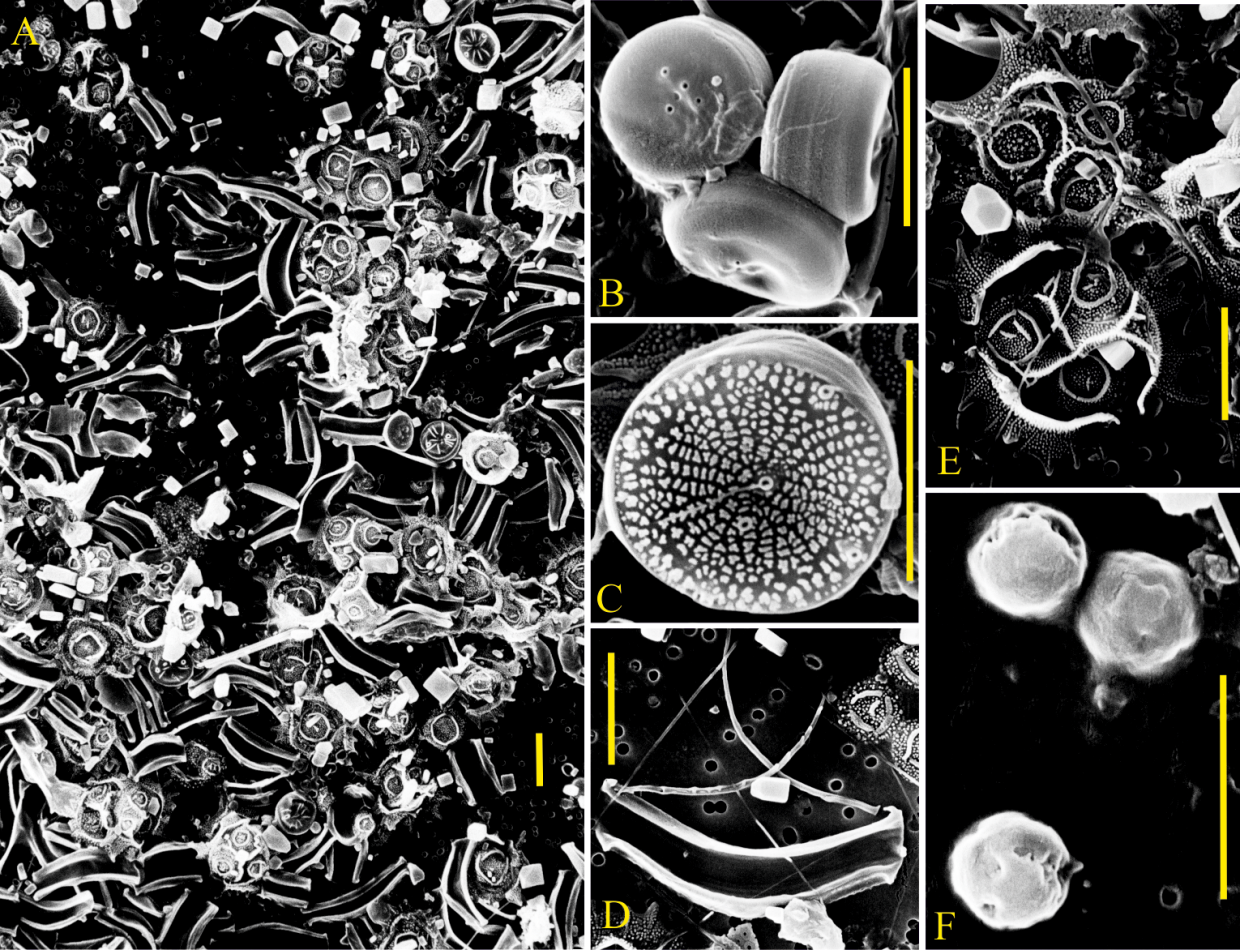
SEM pictures of important pico- and nanophytoplankton species during the two major phytoplankton blooms. A) Representative overview picture from M1 on day 35 including *Arcocellulus* sp., *Minidiscus* sp., and *Tetraparma* sp. (all three are silicifying species). B) Three *Minidiscus* sp. cells without organic membrane cover in M6 on day 35. C) *Minidiscus* sp. without organic membrane cover in M4 on day 27. D) *Arcocellulus* sp. in M1 on day 27 E) Two *Tetraparma* sp. cells in M1 on day 35. F) Three spherical cells (probably picophytoplankton) in M1 on day 35. Yellow scale bars are 3 μm long.

Initially, picoeukaryotes were abundant (Fig 3) and contributed about half of the total chla concentration (Figs 4 and 5). Their abundance started declining, however, around day 10 (Fig 3B and 3J) which is also reflected in a slight decline in chl*a* (Fig 2). The major spring bloom forming groups distinguished by flow cytometry and filter counts were Nano I–IV, Crypto, and *C*. *concinnus*. Their abundances were very low at the beginning but they grew exponentially from the first days until the peak of the bloom around day 33. This is difficult to see on linear cell abundance plots (Fig 3A–3H), but becomes clearly visible when using a logarithmized y-axis (Fig 3I–3P). HPLC pigment measurements and CHEMTAX analysis revealed that diatoms were the dominant taxon during the first bloom (Figs 4 and 5). The bloom-forming diatom community was composed of small nanoplankton species such as *Minidiscus* sp. and *Arcocellulus* sp. (~2–7 μm; Fig 6) and the large mesophytoplankton species *C*. *concinnus* (>200 μm). The bimodal diatom size spectrum with only very small and a very large species is unusual for the study region and will be addressed specifically in a separate paper. Nano I–IV and Crypto abundances decreased rapidly after day ~33 at the end of phase II and dropped to values that were close to those before the first bloom (Fig 3C–3G). This is in contrast to *C*. *concinnus* abundances which showed a less pronounced decrease after the first bloom and recovered quickly thereafter (decrease from ~300–~150 cells L^-1^; Fig 3H).

Phytoplankton groups identified by flow cytometry that markedly participated in the second major bloom in phase III were Peuks, Nano I, and Nano II (Fig 3). Abundances of Cryptos, Nano III, and Nano IV, important groups during the first bloom, remained close to detection limit. Diatoms were also the dominant taxon during the second bloom and represented primarily by *C*. *concinnus*, which was present at similar abundances as during the first bloom (Fig 3). Very small diatoms such as *Arcocellulus* sp. and *Minidiscus* sp. and small silicifying Chrysophyceae (Fig 6) were also present during the second bloom but their biomass contribution was probably lower compared to the first bloom (see also section 4.2.1). Prasinophyceae were the only other noteworthy taxon that contributed to chl*a* but already much less important than diatoms (in average 14% was contributed by Prasinophyceae vs. 82% contributed by diatoms on day 55; Fig 5). The decline of the second bloom towards the end of phase III was reflected in decreasing Peuk, Nano I, Nano II, and *C*. *concinnus* abundances (Fig 3). The observed increases of *Synechococcus* and Crypto abundances during this time were too low to have a predominant influence on the decreasing chl*a* trend that was triggered by the loss of the other groups. During the decline of the second bloom, the community started to shift away from one dominated by diatoms, to a more diverse community. (Fig 5).

The tendency towards a more diverse phytoplankton community continued in phase IV (the post bloom phase) where Prasino-, Crypto-, Cyano-, Chloro-, Prymnesio-, and Dinophyceae became more important (Fig 5). Notably, auto- and/or mixotrophic Dinophyceae were quasi absent during the two diatom-dominated blooms but emerged quickly thereafter (phase IV, Fig 4). The marked increases of Cyano- and Cryptophyceae in phase IV that was revealed by the CHEMTAX analysis was reflected in the increase of *Synechococcus* (a Cyanophyceae genus) and Crypto groups measured with the flow cytometer (compare Figs 3A and 4D). The high consistency among both independent methods increases the confidence in our results.

### 3.3 CO_2_ effects on the phytoplankton community

CO_2_ significantly influenced the development of chl*a* (Table 1), however not consistently. An effect of CO_2_ was absent during the first chl*a* peak in phase II but clearly identifiable during the second bloom in phase III. Here, chl*a* build-up was significantly amplified under high CO_2_ conditions (Fig 2). A shift in the timing (i.e. temporal occurrence) of chl*a* peaks was not apparent. Thus, our results point towards a type A response of chl*a* (increase in bloom amplitude; Fig 1A) during the second phytoplankton bloom in phase III.

**Table 1:** Summary of statistical results. The temporal development of phytoplankton was analyzed by means of GAMM. A CO_2_ effect was detected when the GAMM model with the best fit (highest R^2^ value) accounted for a CO_2_ dependency of the phenology. In the case of Nano I a CO_2_ effect was detected by the GAMM analysis but not considered further due to an unsatisfactory model fit.

analysis	measurement	dependent variable	CO_2_ effect detected?	R^2^ adjusted	most likely response scenario?	remark
GAMM	HPLC	chlorophyll *a*	yes	0.73	Type A	
GAMM	flow cytometry	*Synechococcus*	yes	0.67	Type A	
GAMM	flow cytometry	Peuks	yes	0.71	Type A	
GAMM	flow cytometry	Nano I	(yes)			poor fit of the data
GAMM	flow cytometry	Nano II	no	0.49		
GAMM	flow cytometry	Nano III	no	0.44		
GAMM	flow cytometry	Crypto	no	0.79		
GAMM	flow cytometry	Nano IV	yes	0.54	Type A	
GAMM	filter counts	*C*. *concinnus*	yes	0.83	Type A	
GAMM	HPLC (CHEMTAX)	Diatoms	yes	0.74	Type A	
GAMM	HPLC (CHEMTAX)	Prasinophyceae	yes	0.61	Type A	
GAMM	HPLC (CHEMTAX)	Cryptophyceae	no	0.54		
GAMM	HPLC (CHEMTAX)	Cyanophytceae	yes	0.45	Type A	
GAMM	HPLC (CHEMTAX)	Chlorophyceae	yes	0.3	Type A or C	
GAMM	HPLC (CHEMTAX)	Dinophyceae	yes	0.67	Type B	
GAMM	HPLC (CHEMTAX)	Chrysophyceae	no	0.59		
GAMM	HPLC (CHEMTAX)	Prymnesiophyceae	yes	0.68	Type A	

The GAMM analyses revealed temporal CO_2_ effects in 6 of the 8 taxonomic phytoplankton groups distinguished with CHEMTAX (Table 1). Diatom, Prasinophyceae, and Chlorophyceae biomass was significantly higher under high CO_2_ (Table 1). The positive effect on diatoms occurred for a relatively short period during the second phytoplankton bloom in phase III, similar to bulk chl*a* (compare Figs 2 and 4A). Prasinophyceae were stimulated during a minor peak in phase I and throughout phase III (Fig 4B). Chlorophyceae were close to detection limit during most of the experiment but showed a positive response to high CO_2_ during a peak in phase IV (Fig 4E). Auto- and/or mixotrophic Dinoflagellates (Dinophyceae) experienced positive CO_2_ effects during the end of phases II and IV. Prymnesiophyceae were impaired by high CO_2_ from the end of phase II until the middle of phase IV. (Table 1, Fig 4H). Cyanophyceae were negatively affected under high CO_2_ during phase III and positively affected during phase IV (Table 1), although it must be recognized that the effects were very small and Cyanophyceae were close to detection limit during phase III (Fig 4D).

The GAMM analyses of flow cytometry and filter count data revealed significant temporal CO_2_ effects on 5 out of 8 groups (Table 1) with the clearest CO_2_ response observed for Peuks (Fig 3). Importantly, Peuk abundance was already significantly higher by about 9% (~1500 cells mL^-1^; t-test p = 0.0018) in the high CO_2_ treatment on the first day of the experiment and thus already before the first CO_2_ addition [12]. The reason for this was a carry-over effect from a failed OA experiment we carried out in the same mesocosms before our successful experiment started (see section 2.1). In this previous experiment we already observed a positive CO_2_ effect on picoeukaryotes [12]. However, due to technical problems we had to finish this experiment, lower the mesocosms below surface and dismount the sediment traps until we could restart four days later (section 2.1; [12]). In the four days in between the two studies, water exchange with the fjord was almost but not entirely complete so that some of the CO_2_-induced picoeukaryote signal was transferred into the successful second experiment that is described in the present paper. The small initial difference was lost at the end of phase I (day 17) where abundances in the control and the high CO_2_ treatment were insignificantly different. The first CO_2_ effect on picoeukaryotes that developed during the second experiment started to appear right at the peak of the first chl*a* bloom (day ~33). At this time, Peuk net growth was positive under high CO_2_ and slightly negative in the control mesocosms (Fig 3B and 3J). Opposite net growth rates were observed until day ~47 and generated an offset in Peuk abundance between control and high CO_2_ mesocosms which prevailed until the end of phase III (Fig 3B and 3J). A second divergence in Peuk abundance occurred during phase IV where they bloomed under high CO_2_ (~38,000 cells mL^-1^ on day 91) but not in the control (Fig 3B and 3J).

The abundance of *C*. *concinnus* was significantly elevated under high CO_2_, mainly during the second phytoplankton bloom in phase III (Fig 3H and 3P). *Synechoccocus* abundance was slightly lower under high CO_2_ during phase III and marginally higher during a short period in phase IV (Fig 3I, Table 1). Nano IV abundance was lower under high CO_2_ during the beginning of phase IV but the effect was very small (Fig 3O). The detected CO_2_ effect on Nano I (Table 1) must be regarded carefully. Here, short consecutive abundance peaks constrained the generation of adequate GAMM fits (S2 Fig) so that a reliable determination of CO_2_ effects was not possible.

## 4. Discussion

### 4.1 The potential influence of ocean acidification on phytoplankton blooms

We observed no detectable CO_2_ effect on chl*a* during the first bloom in phase II (Fig 2) where phytoplankton utilized inorganic nutrients that were initially available from winter upwelling. This outcome is consistent with results from the majority of previous mesocosm OA experiments under nutrient replete conditions. So far, ten studies with mesocosm volumes ≥ 100 L reported no response of CO_2_ on maximum chl*a* build-up [28–37], while only five detected either positive [35,37–39] or negative [40] impacts.

A positive CO_2_ effect on chl*a* build-up was observed during the second bloom in phase III. The CO_2_ effect did not appear to be particularly pronounced (Fig 2) but this may not reflect the actual chl*a* difference appropriately because we also observed significantly higher mesozooplankton biomass under high CO_2_ during this period [20,27]. Thus, part of the chl*a* difference may have been grazed off.

Inorganic nutrient concentrations were close to detection limit during the second bloom so that the bloom was fueled by other nutrient sources. Published results on mesocosm OA experiments conducted under inorganic nutrient deplete conditions are less numerous so far, making it even more difficult to reveal a general response pattern. The seven mesocosm experiments we are aware of (volume ≥ 100 L; published until June 2017) either observed a stimulation of chl*a* concentrations under high CO_2_ [35,41] or reported no effect [37,39,42]. Accordingly, the chl*a* response pattern observed in our study aligns reasonably well with the general tendency currently taking form in the literature—i.e. rather no chl*a* response to OA under nutrient replete conditions and perhaps a slight tendency towards a positive response when inorganic nutrients are low [42]. However, more data and thorough meta-analyses that consider the individual features of experiments are needed to confirm or disprove this impression.

### 4.2 CO_2_ effects in the phytoplankton community

CO_2_ effects on individual phytoplankton groups were identified in 10 out of 16 parameters analyzed with GAMM (Table 1; Nano I was not considered here). As for chl*a*, most of the detected effects were present only during certain stages of the succession and effect sizes appeared to be small in most cases (Figs 3 and 4). Uncovering the origin of group-specific CO_2_ responses is challenging in ecologically realistic experiments because the multitude of unconstrained factors allows for a multitude of potential explanations. For example, changing CO_2_ or pH can lead to a direct (i.e. physiological) response of the investigated taxon with direct consequences for its competitiveness within the natural community. In this case, results from physiological laboratory investigations can be used to explain certain patterns. However, observed responses can equally well be evoked indirectly, via CO_2_ effects on other players in the food web which influence the investigated taxon through trophic cascades. Indirect effects are considered to be very important but hard to prove as they require a comprehensive understanding of the various interactions in the food web [43]. In the following, we aim to present what we consider to be the most likely explanations for the observed CO_2_ responses in some of the investigated phytoplankton groups. We would like to emphasize, however, that explanations different to the ones provided here are possible in each case.

#### 4.2.1 Diatoms

Diatoms were dominating the phytoplankton community and their temporal development is largely identical to the development of bulk chl*a*. (compare Figs 2 and 4A) This suggests that the positive CO_2_ effect on chl*a* during phase III is primarily a positive CO_2_ effect on diatoms. Diatoms were represented by very small species (~2–8 μm) such as *Minidiscus* sp. or *Arcocellulus* sp. and by the large species *C*. *concinnus* (>200 μm). The abundance of *C*. *concinnus* was significantly higher in the high CO_2_ treatment by about 70 cells L^-1^ during the peak of the second bloom in phase III (average between days 45 to 55 of 255 and 324 cells mL^-1^ in the control and the high CO_2_ mesocosms, respectively). To approximate the relevance of this difference in terms of chl*a*, we measured chl*a* content of *C*. *concinnus* cells on days 45 and 49, multiplied the average chl*a* cell^-1^ value with measured cell numbers, and compared this to bulk chl*a* concentrations. Based on that, *C*. *concinnus* contributed about 50% (ranging from 36% in M5 to 66% in M4) to the bulk chl*a* concentration during phase III. The difference of ~70 cells L^-1^ between control and high CO_2_ explains about half (i.e. 0.5 μg L^-1^) of the CO_2_-induced difference in bulk chl*a* (i.e. ~1 μg L^-1^; Fig 2). The CHEMTAX diatom trend suggests, however, that the entire 1 μg L^-1^ difference is due to differences in diatom biomass (compare Figs 2 and 4A). Thus, the remaining 0.5 μg L^-1^ must have been due to biomass differences in the small diatom species like e.g. *Arcocellulus* sp. (Fig 6). Unfortunately, there is no biomass data on any of the small diatoms available but due to their approximate size they must have been included in the Nano I and/or Nano II populations quantified with the flow cytometry. Here, we do not find any CO_2_-related differences (Fig 3) meaning that the CHEMTAX and the flow cytometry data are conflicting in this particular case. We found no explanation for this other than uncertainties in the associated measurements and the abovementioned biomass estimation of *C*. *concinnus*.

The elevated *C*. *concinnus* abundances observed in the high CO_2_ treatment occurred during phase III where inorganic nutrients were depleted. This is consistent with results from a recent laboratory study where the diatoms *Thalassiosira weissflogii* and *Dactyliosolen fragilissimus* also reached higher population densities under high CO_2_ when nutrients were exhausted [44]. The authors hypothesized that less resources were necessary for inorganic carbon acquisition under high CO_2_ thereby allocating resources to growth which leads to higher population densities [44]. Interestingly, CO_2_ stimulation was shown to be much more pronounced in larger diatoms as these are considered to be more diffusion limited [44–46]. This may explain why we found a clear positive CO_2_ effect in the large (i.e. >200 μm) diatom *C*. *concinnus*.

The line of reasoning presented above points towards a direct (i.e. physiological) effect of CO_2_ on the growth of *C*. *concinnus*. An indirect effect through food web interactions seems less likely, also because *C*. *concinnus* was too large to be grazed by any of the present zooplankton species [20] including herring larvae where no *C*. *concinnus* was found in the gut content. Thus, our results support the hypothesis that large diatom genera like *Coscinodiscus* could become more competitive in an acidified ocean under nutrient deplete conditions through facilitated inorganic carbon acquisition [44,45]. In contrast, our observations on small diatoms are inconclusive, mainly because our data is not resolved with the necessary detail on diatom community structure in the small size range.

#### 4.2.2 Dinoflagellates

Dinoflagellates are a diverse group of protists which acquire energy through photo- or heterotrophy or a combination of both known as mixotrophy [47]. Here, we determined dinoflagellate contribution to chl*a* with CHEMTAX and therefore only considered photosynthesizing species with a pigment setup characteristic for Dinophyceae [16]. This excludes heterotrophic species and mixotrophic ones which acquire plastids from other phytoplankton taxa (e.g. *Dinophysis* which sequesters cryptophyceae chloroplasts from its prey [47]). Dinophyceae were growing early in the experiment but started to decline at the beginning of phase II in the control mesocosms. High CO_2_ did not affect the maximum biomass but delayed their decline by two weeks (Fig 4F). Based on microscopy counts we identified *Heterocapsa triquetra* as the most likely species responsible for the observed trends in the CHEMTAX data during phase I and II since it was the only dinoflagellate species found in noticeable quantities during this time. *H*. *triquetra* is primarily phototrophic but can apply phagotrophy under nutrient-limiting conditions to acquire nitrogen and phosphorous [48]. Culture experiments suggested that the growth rate of *H*. *triquetra* is unaffected by pH in the range between 8.7–7.5 [49]. This argues against a direct CO_2_ effect on *H*. *triquetra* growth rate and points towards an indirect effect, for example through reduced grazing pressure under high CO_2_ during phase II.

Dinophyceae were not detected for most of phase III but started to increase again during phase IV. They reached higher biomass in the high CO_2_ treatment towards the end of the experiment (Fig 4F). Horn et al., investigated dinoflagellate abundance in the same mesocosm study by means of light microscopy and found the same CO_2_ trend in phase IV [50]. In their analysis they focused on species which are traditionally considered as heterotrophic although still aware that many species are at least facultative mixotrophic [50]. The CO_2_ effect detected by Horn et al. was caused by elevated abundances of athecate dinoflagellates (<30–55 μm) [50] represented primarily by *Gyrodinium* and/or *Gymnodinium* sp. (H. Horn, pers. comm.). The authors hypothesized that the positive CO_2_ effect on these mixotrophic species was caused by increased availability of picoeukaryote (Peuk) prey [50]. Our data supports this hypothesis since Peuk abundance was indeed elevated under high CO_2_ before the onset of the second dinoflagellate bloom and then rapidly declined to very low numbers when Dinophyceae started to grow (Fig 4). The elevated availability of picoeukaryotic prey under high CO_2_ may have enabled Dinophyceae to reach higher biomass on the last days of the experiment.

#### 4.2.3 Prymnesiophyceae

Prymnesiophyceae had a minor contribution to total chl*a* (Fig 5). Their biomass peaked in the aftermath of the two major phytoplankton blooms and was lower in the high CO_2_ treatment throughout almost the entire experiment (Fig 4H). A recent synthesis of OA studies with natural plankton communities found a consistently negative CO_2_ effect on Prymnesiophyceae (aka Haptophyceae) biomass with only few exceptions [7]. Negative effects were often driven by calcifying Prymnesiophyceae (coccolithophores) [7], which are known to be sensitive to low pH [51,52]. However, non-calcifying genera like *Phaeocystis* or *Chrysochromulina* also responded negatively to increasing CO_2_ [7]. Unfortunately, we were unable to identify the species or species assemblage causing the negative CO_2_ response in our study but the high consistency among the various mesocosm experiments with taxonomically very different Prymnesiophyceae species points towards a physiological carbonate chemistry sensitivity that is rooted in the core physiological apparatus of this taxon. An (indirect) CO_2_ effect on Prymnesiophyceae through food web interactions seems rather unlikely because in this case we would have expected a more variable response among previous studies and also a less consistent negative CO_2_ effect in our “long-term” study. Collectively, the evidence from multiple experiments suggests that Prymnesiophyceae face the risk of playing a less important role in plankton communities in an acidified ocean.

#### 4.2.4 Picocyanobacteria (Cyanophyceae)

Picocyanobacteria were present throughout the entire study although they played a minor role in terms of biomass and occurred in high abundances only at the end of the experiment (Figs 3A and 5). They were represented most likely by the genus *Synechococcus* and not *Prochlorococcus* because the latter is not occurring above 40°N [53] and its marker pigments (divinyl chlorophyll *a* and *b* [54]) were not detected. The temporal development of *Synechococcus* counted with the flow cytometer, and Cyanophyceae, determined with CHEMTAX agree well with each other (compare Figs 3A and 4D) suggesting that *Synechococcus* was the only cyanobacterium genus present in noticeable amounts. CO_2_ had a weakly negative effect on its abundance (and Cyanophyceae biomass) during phase III and a marginally positive one during phase IV (Figs 3A and 4D). Previous experiments with pelagic communities revealed variable responses of *Synechococcus* abundances to simulated OA (positive, negative, neutral) which was attributed to the enormous cryptic diversity of this genus [7,55,56].

Alternatively, indirect CO_2_ effects could explain their variable responses. In our experiment, the negative CO_2_ effect manifested shortly after inorganic nutrients were exhausted (~day 33) and the major spring bloom was on the decline (Fig 2; phase II). We observed no significant CO_2_ effect on predominant microzooplanktonic grazers such as ciliates and heterotrophic dinoflagellates during this period [50] but detected a positive effect on picoeukaryotes appearing precisely when *Synechococcus* responded negatively to CO_2_ (Fig 3). Indeed, picoeukaryote genera like *Micromonas* can be mixotrophic and feed on spherical particles with a size of at least 0.9 μm in diameter [57–59]. Thus, enhanced grazing on *Synechococcus* by picoeukaryotes under high CO_2_ could potentially explain their negative CO_2_ response during phase III (mixotrophy of picoeukaryotes is discussed further in section 4.2.5; please note that Dinophyceae, represented by the mixotroph *H*. *triquetra* (section 4.2.2), also respond positively to CO_2_ during this time but this species does not feed on *Synechococcus* [48]). A similar antagonistic CO_2_ response between picoeukaryotes and *Synechococcus* has also been observed in a previous mesocosm study in Raunefjord (Norway) [60]. Here, Paulino et al. speculated that picoeukaryotes were better nutrient competitors under high CO_2_ relative to *Synechococcus* [60]. Findings by Paulino et al. are in contrast to the findings by Schulz et al. who observed a synergistic response of picoeukaryotes and *Synechococcus* in a follow-up OA mesocosm experiment at the Raunefjord study site (both Synechococcus and picoeukaryote abundance was stimulated by high CO_2_ [7]). However, in the case of the Schulz et al study, picoeukaryotes were dominated by Chlorophyceae whereas Paulino et al. argue that they were dominated by Prasinophyceae (*Micromonas*) in their particular study [7,60]. In accordance with both studies, we observed an antagonistic response during phase III where picoeukaryotes were dominated by Prasinophyceae whereas a synergistic response occurred during phase IV where picoeukaryotes were predominantly composed of Chlorophyceae (see section 4.2.5). Thus, CO_2_ effects on *Synechococcus* may be coupled to the taxonomic composition and the trophic interactions with their picoeukaryotic competitors.

#### 4.2.5 Picoeukaryotes (Prasinophyceae and Chlorophyceae)

The abundance of picoeukaryotes (Peuks) was positively affected by high CO_2_ at different stages of the winter-to-summer succession. The Peuk clusters determined by means of flow cytometry were most likely dominated by Prasino- and Chlorophyceae as the combined pattern closely resembles the Peuks trend over time (compare Figs 3B with 4B and 4E; see also section 4.2.4). This is in line with previous studies who also determined Prasino- and Chlorophyceae as the predominant picoeukaryotes [7].

The small but significant difference in Peuk abundance between control and high CO_2_ at the first day was a remnant of a preceding CO_2_ experiment (see section 3.3). We cannot fully exclude that this initial difference was also causing the differences observed later in the experiment but several reasons make this unlikely. Most importantly, the difference was small (1500 cells mL^-1^; less than 9% of the population) and could be equalized quickly under the assumption of realistic picoeukaryote growth rates [12]. Indeed, mean Peuk abundances equalized between control and high CO_2_ already quite early in the experiment and it lasted more than two weeks until deviating Peuk abundances between control and high CO_2_ treatment reestablished (day ~33; Fig 3B and 3J). We would have expected a continuous offset between control and high CO_2_ rather than a reoccurring one in case the initial difference was responsible for the deviating trends later in the experiment. Furthermore, Peuks belonged primarily to the Prasinophyceae class at the beginning of the study while a large fraction belonged to the Chlorophyceae at a later stage. It seems rather unlikely that an initial difference in one class triggered the same response in another class later in the experiment. To conclude this line of arguments we would like to point out the following: Even in the unlikely case that the positive CO_2_ responses of Peuks observed in this study were triggered by the small initial difference, our interpretations would still be valid. This is because the initial difference itself is not a coincidence but the result of a positive CO_2_ effect on Peuks occurring in the preceding experiment which was stopped due to technical problems (section 2.1; [12]).

Stimulation of phytoplankton growth rate and abundance by elevated levels of CO_2_ has frequently been observed in cell cultures and natural assemblages [61]. The phenomenon is typically explained by a CO_2_ fertilization of the often rate-limited carbon fixing enzyme Rubisco [62]. This straight-forward hypothesis may also be true for picoeukaryotes where *in-vitro* experiments documented accelerated growth rates of important picoeukaryote genera like *Ostreococcus* and *Micromonas* under above ambient pCO_2_ (i.e. ~500–1000 μatm; [63,64]). It is surprising, however, that we found a particularly pronounced CO_2_-stimulation on abundance in the smallest eukaryotic phytoplankton group. In theory, we would expect that primarily larger species like *C*. *concinnus* benefit more from high CO_2_ because they are more diffusion-limited due to their lower surface to volume (S/V) ratio (section 4.2.1; [46,65]). This counter-intuitive result indicates that additional (or complementary) mechanisms may have determined the specific stimulation of Peuks. We propose three of these mechanisms in the following. All three are related to nutrient acquisition when inorganic nutrients are limiting.

1. The largest differences in Peuk net growth between control and high CO_2_ were observed after day 33 when NO_3_^-^+NO_2_^-^ concentrations were close to detection limit (days ~33–47 and ~80–90 in Fig 3J; nutrient concentrations are shown in S3 Table). These conditions primarily select for phytoplankton with high abilities to gather nutrients from the environment [66]. In general, smaller phytoplankton groups are considered to be more capable in nutrient acquisition than larger ones due to their relatively high S/V ratio [67]. Thus, Peuks may be the phytoplankton group who could capitalize best on the CO_2_ fertilization of photosynthesis under nutrient-limiting conditions since they were superior nutrient competitors.
2. The second mechanism follows the same underlying logic as described in the first one but takes a pH dependency of remineralization rather than a CO_2_ dependency of photosynthesis into account. Previous OA studies with auto- and heterotrophic bacteria reported accelerated rates of extracellular enzymes involved in organic matter remineralization under low pH [68–72]. If the same pH dependency also applies for eukaryotic phytoplankton, they should have an advantage in the extraction of nutrients from organic sources under acidified conditions. This would be once more particularly beneficial for picoeukaryotes due to their increased S/V ratio relative to larger species.
3. The third mechanism we propose is related to the mixotrophic abilities of picoeukaryotes. Recent field studies revealed the potential of photosynthesizing picoeukaryotes to satisfy part of their nutrient requirements through phagocytosis of bacteria in oligotrophic regimes [58,59,73]. CO_2_ fertilization of photosynthesis in the OA treatment may have raised the nutrient requirements of Peuks and therefore stimulated grazing on bacteria. This hypothesis is supported by the abundance ratio of Peuks to heterotrophic bacteria which was significantly elevated under high CO_2_ during the picoeukaryote bloom under inorganic nutrient deplete conditions in phase III (S3 Fig). This antagonistic pattern was also observed in a previous mesocosm study in oligotrophic post-bloom conditions [74]. It has been hypothesized that bacterivory by mixotrophs can serve as alternative nutrient source when inorganic nutrients are limiting and simultaneously weaken heterotrophic bacteria as nutrient competitors [75]. Indeed, laboratory experiments with mixotrophic phytoplankton showed that photosynthesizing cells can adjust phagotrophic rates to changing nutrient concentration and/or light intensity in order to sustain optimal nutrient supply [48,76]. Thus, it is possible that such an adjustment of phagotrophic rates also occurs when the nutrient demand of Peuks is altered by changing carbonate chemistry.

Another noticeable positive CO_2_ effect on Peuk abundance was observed later in the experiment during phase IV (around day 90; Fig 3J). This Peuk bloom was different to the previous ones in that it was dominated by Chlorophyceae and not Prasinophyceae. Accordingly, the positive CO_2_ effect on picoeukaryotes seems to be related to their size and their role in the food web rather than on their taxonomic classification.

The positive effect of end-of-the-century CO_2_ partial pressures on picoeukaryote abundance is a strikingly consistent result in ocean acidification studies with plankton communities [7]. This has been shown from eutrophic to oligotrophic regimes [41,60], from high to lower latitudes [35,37,77], from winter to summer [this study], and from marine to freshwater environments [78,79]. Other climate change related consequences such as ocean warming, freshening, and enhanced stratification also seem to favor picoeukaryotes [80–83]. Thus, multiple evidences from different studies and different climate change related drivers strongly suggest that the proliferation of picoeukaryotes in the future ocean is likely.

## Supporting information

S1 Table Pigment to chlorophyll *a* (chl*a*) ratios of input (F0) and output (F1) matrices from the CHEMTAX analysis. Chlorophyll c3/chl*a* (chlc3), Chlorophyll c2/chl*a* (chlc2), Peridinin/chl*a* (Peri), 19-Butanoyloxyfucoxanthin/chl*a* (19-But), Fucoxanthin/chl*a* (Fuco), Neoxanthin/chl*a* (Neox), Prasinoxanthin/chl*a* (Prasino), Violaxanthin/chl*a* (Viola), 19-Hexanoyloxyfucoxanthin/chl*a* (19-Hex), Diadinoxanthin/chl*a* (Diadino), Alloxanthin/chl*a* (Allox), Diatoxanthin/chl*a* (Diatox), Lutein/chl*a* (Lutein), chlorophyll b/chl*a* (chlb).
(XLSX)

S2 Table List of species identified by means of light microscopy. (XLSX)

S3 Table Average inorganic nutrient concentrations during each phase. The inorganic nutrient development is was similar in all mesocosms [12] so that the averages shown here include all mesocosms. A graphical representation of the inorganic nutrient dataset as well as the analytical methodology is provided in the overview paper [12] accompanying this study. Phase I = day -2–16; phase II = day 17–40; phase III = day 41–77; phase IV = day 78–111.
(XLSX)

S1 Fig Gating strategy in the flow cytometer analysis. Plots A–C and E–G show the gates for Peuks and Nano I–IV in mesocosm 4 (A = day -1, B = day 35, C = day 93) and mesocosm 10 (E = day -1, F = day 35, G = day 93). Please note that gates were adjusted in the course of the experiments to account for changing population appearances (section 2.2). Plots D and H show the gates of *Synechococcus* and Crypto populations, respectively. These gates remained unchanged during the entire study.
(PDF)

S2 Fig Generalized additive mixed-effect model (GAMM) results. The blue and red lines are fitted GAMMs with the shaded areas representing confidence intervals. CO_2_ effects were detected when both a red and a blue line are present in the plots. A blue line is always present meaning that time always had a significant effect on the trends. Blue and red dots are underlying raw data from 5 control and 5 high CO_2_ mesocosms, respectively. A summary on the GAMM results is provided in Table 1.
(TIFF)

S3 Fig Development of picoeukaryotes and bacteria abundance relative to each other. Red and blue lines show the average of five high and five ambient CO_2_ mesocosms, respectively. Shaded areas represent standard deviations from means. Vertical grey lines (Roman numbers I to IV) separate the four experimental phases. (A) Peuk abundance (same as in Fig 3B). (B) Bacteria abundance. (C) Peuk to bacteria abundance ratio. Statistical significance was detected in all three datasets by means of GAMM (Peuk abundance R^2^_adj._ = 0.71, bacteria abundance R^2^_adj._ = 0.72, Peuk/bacteria ratio R^2^_adj._ = 0.76).
(TIF)

## References

[1] Sommer U, Adrian R, De Senerpont Domis L, Elser JJ, Gaedke U, Ibelings B, et al Beyond the Plankton Ecology Group (PEG) Model: Mechanisms Driving Plankton Succession. Annu Rev Ecol Evol Syst. 2012;43: 429–448. doi: 10.1146/annurev-ecolsys-110411-160251

[2] Behrenfeld MJ. Abandoning Sverdrup’s Critical Depth Hypothesis on phytoplankton blooms. Ecology. 2010;91: 977–989. doi: 10.1890/09-1207.1 20462113 10.1890/09-1207.1

[3] Behrenfeld MJ, Boss ES. Resurrecting the ecological underpinnings of ocean plankton blooms. Ann Rev Mar Sci. 2014;6: 167–194. doi: 10.1146/annurev-marine-052913-021325 24079309 10.1146/annurev-marine-052913-021325

[4] Margalef R. Life-forms of phytoplankton as survival alternatives in an unstable environment. Oceanol Acta. 1978;1: 493–509.

[5] Lampert W, Fleckner W, Rai H, Taylor BE. Phytoplankton control by grazing zooplankton: A study on the spring clear-water phase. Limnol Oceanogr. 1986;31: 478–490. doi: 10.4319/lo.1986.31.3.0478

[6] Tortell PD, DiTullio GR, Sigman DM, Morel FMM. CO_2_ effects on taxonomic composition and nutrient utilization in an Equatorial Pacific phytoplankton assemblage. Mar Ecol Prog Ser. 2002;236: 37–43. doi: 10.3354/meps236037

[7] Schulz KG, Bach LT, Bellerby R, Bermudez R, Boxhammer T, Czerny J, et al Phytoplankton blooms at increasing levels of atmospheric carbon dioxide: experimental evidence for negative effects on prymnesiophytes and positive on small picoeukaryotes. Front Mar Sci. 2017;4: 1–18. doi: 10.3389/fmars.2017.00064

[8] Rost B, Zondervan I, Wolf-Gladrow D. Sensitivity of phytoplankton to future changes in ocean carbonate chemistry: Current knowledge, contradictions and research directions. Mar Ecol Prog Ser. 2008;373: 227–237. doi: 10.3354/meps07776

[9] Hoppe CJM, Hassler CS, Payne CD, Tortell PD, Rost BR, Trimborn S. Iron limitation modulates ocean acidification effects on Southern Ocean phytoplankton communities. PLoS One. 2013;8 doi: 10.1371/journal.pone.0079890 24278207 10.1371/journal.pone.0079890 PMC3835797

[10] Riebesell U, Bach LT, Bellerby RGJ, Monsalve JRB, Boxhammer T, Czerny J, et al Competitive fitness of a predominant pelagic calcifier impaired by ocean acidification. Nat Geosci. 2017;10: 19–23. doi: 10.1038/NGEO2854

[11] Dutkiewicz S, Morris JJ, Follows MJ, Scott J, Levitan O, Dyhrman ST, et al Impact of ocean acidification on the structure of future phytoplankton communities. Nat Clim Chang. 2015;5: 1002–1006. doi: 10.1038/nclimate2722

[12] Bach LT, Taucher J, Boxhammer T, Ludwig A, Achterberg EP, Algueró-Muñiz M, et al Influence of Ocean Acidification on a Natural Winter-to-Summer Plankton Succession: First Insights from a Long-Term Mesocosm Study Draw Attention to Periods of Low Nutrient Concentrations. PLoS One. 2016;11: e0159068 doi: 10.1371/journal.pone.0159068 27525979 10.1371/journal.pone.0159068 PMC4985126

[13] Riebesell U, Czerny J, von Bröckel K, Boxhammer T, Büdenbender J, Deckelnick M, et al Technical Note: A mobile sea-going mesocosm system—new opportunities for ocean change research. Biogeosciences. 2013;10: 1835–1847. doi: 10.5194/bg-10-1835-2013

[14] Boxhammer T, Bach LT, Czerny J, Riebesell U. Technical Note: Sampling and processing of mesocosm sediment trap material for quantitative biogeochemical analysis. Biogeosciences. 2016;13: 2849–2858. doi: 10.5194/bgd-12-18693-2015

[15] Barlow RG, Cummings DG, Gibb SW. Improved resolution of mono- and divinyl chlorophylls a and b and zeaxanthin and lutein in phytoplankton extracts using reverse phase C-8 HPLC. Mar Ecol Prog Ser. 1997;161: 303–307. doi: 10.3354/meps161303

[16] Mackey MD, Mackey DJ, Higgins HW, Wright SW. CHEMTAX- a program for estimating class abundances from chemical markers: application to HPLC measurements of phytoplankton. Mar Ecol Prog Ser. 1996;144: 265–283.

[17] Utermöhl T. Zur Vervollkommnung der quantitativen Phytoplankton-Methodik. Vereinigung für Theor und Angew Limnol. 1958;9: 1–38.

[18] Bach LT, Bauke C, Meier KJS, Riebesell U, Schulz KG. Influence of changing carbonate chemistry on morphology and weight of coccoliths formed by *Emiliania huxleyi*. Biogeosciences. 2012;9: 3449–3463. doi: 10.5194/bg-9-3449-2012

[19] Olson RJ, Zettler ER, Anderson OK. Discrimination of eukaryotic phytoplankton cell types from light scatter and autofluorescence properties measured by flow cytometry. Cytometry. 1989;10: 636–643. doi: 10.1002/cyto.990100520 2776580 10.1002/cyto.990100520

[20] Taucher J, Haunost M, Boxhammer T, Bach LT, Algueró-Muñiz M, Riebesell U. Influence of ocean acidification on plankton community structure during a winter-to-summer succession: An imaging approach indicates that copepods can benefit from elevated CO_2_ via indirect food web effects. PLoS One. 2017;12: e0169737 doi: 10.1371/journal.pone.0169737 28178268 10.1371/journal.pone.0169737 PMC5298333

[21] Veldhuis MJW, Kraay GW. Application of flow cytometry in marine phytoplankton research: current applications and future perspectives. Sci Mar. 2000;64: 121–134.

[22] Marie D, Partensky F, Vaulot D, Brussaard CPD. Enumeration of phytoplankton, bacteria, and viruses in marine samples In: Robinson JPEA, editor. Current protocols in cytometry. New York: John Wiley and sons; 2001 pp. 1–14. doi: 10.1002/0471142956.cy1111s10 10.1002/0471142956.cy1111s10 18770685

[23] Karner MB, DeLong EF, Karl DM. Archaeal dominance in the mesopelagic zone of the Pacific Ocean. Nature. 2001;409: 507–510. doi: 10.1038/35054051 11206545 10.1038/35054051

[24] Wood SN. Fast stable restricted maximum likelihood and marginal likelihood estimation of semiparametric generalized linear models. J R Stat Soc Ser B (Statistical Methodol. 2011;73: 3–36.

[25] Hastie T, Tibshirani R. Generalized additive models. Stat Sci. 1986;1: 297–318. 10.1177/096228029500400302 8548102

[26] Zuur A, Ieno EN, Walker N, Saveliev AA, Smith GM. Mixed Effects Models and Extensions in Ecology with R. New York: Springer-Verlag; 2009 doi: 10.1007/978-0-387-87458-6

[27] Algueró-Muñiz M, Alvarez-Fernandez S, Thor P, Bach LT, Esposito M, Horn HG, et al Ocean acidification effects on mesozooplankton community development: results from a long-term mesocosm experiment. PLoS One. 2017;12: e0175851 doi: 10.1371/journal.pone.0175851 28410436 10.1371/journal.pone.0175851 PMC5391960

[28] Engel A, Zondervan I, Aerts K, Beaufort L, Benthien A, Chou L, et al Testing the direct effect of CO_2_ concentration on a bloom of the coccolithophorid *Emiliania huxleyi* in mesocosm experiments. Limnol Oceanogr. 2005;50: 493–507.

[29] Engel A, Schulz KG, Riebesell U, Bellerby R, Delille B, Schartau M. Effects of CO_2_ on particle size distribution and phytoplankton abundance during a mesocosm bloom experiment (PeECE II). Biogeosciences. 2008;5: 509–521. doi: 10.5194/bgd-4-4101-2007

[30] Engel A, Piontek J, Grossart H-P, Riebesell U, Schulz KG, Sperling M. Impact of CO_2_ enrichment on organic matter dynamics during nutrient induced coastal phytoplankton blooms. J Plankton Res. 2014;36: 641–657. doi: 10.1093/plankt/fbt125

[31] Lindh M V., Riemann L, Baltar F, Romero-Oliva C, Salomon PS, Granéli E, et al Consequences of increased temperature and acidification on bacterioplankton community composition during a mesocosm spring bloom in the Baltic Sea. Environ Microbiol Rep. 2013;5: 252–262. doi: 10.1111/1758-2229.12009 23584969 10.1111/1758-2229.12009

[32] Paul C, Matthiessen B, Sommer U. Warming, but not enhanced CO_2_ concentration, quantitatively and qualitatively affects phytoplankton biomass. Mar Ecol Prog Ser. 2015;528: 39–51. doi: 10.3354/meps11264

[33] Calbet A, Sazhin AF, Nejstgaard JC, Berger S a, Tait ZS, Olmos L, et al Future climate scenarios for a coastal productive planktonic food web resulting in microplankton phenology changes and decreased trophic transfer efficiency. PLoS One. 2014;9: e94388 doi: 10.1371/journal.pone.0094388 24721992 10.1371/journal.pone.0094388 PMC3983207

[34] Riebesell U, Schulz KG, Bellerby RGJ, Botros M, Fritsche P, Meyerhöfer M, et al Enhanced biological carbon consumption in a high CO_2_ ocean. Nature. 2007;450: 545–548. doi: 10.1038/nature06267 17994008 10.1038/nature06267

[35] Sala MM, Aparicio FL, Balagué V, Boras JA, Borrull E, Cardelús C, et al Contrasting effects of ocean acidification on the microbial food web under different trophic conditions. Ices J Mar Sci. 2015;73: 670–679. doi: 10.1093/icesjms/fsv130

[36] Rossoll D, Sommer U, Winder M. Community interactions dampen acidification effects in a coastal plankton system. Mar Ecol Prog Ser. 2013;486: 37–46. doi: 10.3354/meps10352

[37] Thomson PG, Davidson AT, Maher L. Increasing CO_2_ changes community composition of pico- and nano-sized protists and prokaryotes at a coastal Antarctic site. Mar Ecol Prog Ser. 2016;554: 51–69. doi: 10.3354/meps11803

[38] Hopkins FE, Turner SM, Nightingale PD, Steinke M, Bakker D, Liss PS. Ocean acidification and marine trace gas emissions. Proc Natl Acad Sci U S A. 2010;107: 760–765. doi: 10.1073/pnas.0907163107 20080748 10.1073/pnas.0907163107 PMC2818925

[39] Schulz KG, Bellerby RGJ, Brussaard CPD, Büdenbender J, Czerny J, Engel A, et al Temporal biomass dynamics of an Arctic plankton bloom in response to increasing levels of atmospheric carbon dioxide. Biogeosciences. 2013;10: 161–180. doi: 10.5194/bg-10-161-2013

[40] Kim JH, Kim KY, Kang EJ, Lee K, Kim JM, Park KT, et al Enhancement of photosynthetic carbon assimilation efficiency by phytoplankton in the future coastal ocean. Biogeosciences. 2013;10: 7525–7535. doi: 10.5194/bg-10-7525-2013

[41] Paul AJ, Bach LT, Schulz K-G, Boxhammer T, Czerny J, Achterberg EP, et al Effect of elevated CO_2_ on organic matter pools and fluxes in a summer Baltic Sea plankton community. Biogeosciences. 2015;12: 6181–6203.

[42] Gazeau F, Sallon A, Pitta P, Tsiola A, Maugendre L, Giani M, et al Limited impact of ocean acidification on phytoplankton community structure and carbon export in an oligotrophic environment: Results from two short-term mesocosm studies in the Mediterranean Sea. Estuar Coast Shelf Sci. Elsevier Ltd; 2016; 1–17. doi: 10.1016/j.ecss.2016.11.016

[43] Strauss SY. Effects in Community Ecology: Their Definition, Study and Importance. Trends Ecol Evol. 1991;6: 206–210. doi: 10.1016/0169-5347(91)90023-Q 21232460 10.1016/0169-5347(91)90023-Q

[44] Taucher J, Jones J, James A, Brzezinski MA, Carlson CA, Riebesell U, et al Combined effects of CO_2_ and temperature on carbon uptake and partitioning by the marine diatoms *Thalassiosira weissflogii* and *Dactyliosolen fragilissimus*. Limnol Oceanogr. 2015;60: 901–919. doi: 10.1002/lno.10063

[45] Wu Y, Campbell DA, Irwin AJ, Suggett DJ, Finkel Z V. Ocean acidification enhances the growth rate of larger diatoms. Limnol Oceanogr. 2014;59: 1027–1034. doi: 10.4319/lo.2014.59.3.1027

[46] Wolf-Gladrow D, Riebesell U. Diffusion and reactions in the vicinity of plankton: A refined model for inorganic carbon transport. Mar Chem. 1997;59: 17–34. doi: 10.1016/S0304-4203(97)00069-8

[47] Stoecker DK, Hansen PJ, Caron DA, Mitra A. Mixotrophy in the Marine Plankton. Ann Rev Mar Sci. 2017;9: 311–335. doi: 10.1146/annurev-marine-010816-060617 27483121 10.1146/annurev-marine-010816-060617

[48] Legrand C, Granéli E, Carlsson P. Induced phagotrophy in the photosynthetic dinoflagellate Heterocapsa triqueta. Aquat Microb Ecol. 1998;15: 65–75. doi: 10.3354/ame015065

[49] Berge T, Daugbjerg N, Balling Andersen B, Hansen P. Effect of lowered pH on marine phytoplankton growth rates. Mar Ecol Prog Ser. 2010;416: 79–91. doi: 10.3354/meps08780

[50] Horn HG, Sander N, Stuhr A, Algueró-Muñiz M, Bach LT, Löder MGJ, et al Low CO_2_ sensitivity of microzooplankton communities in the Gullmar Fjord, Skagerrak: Evidence from a long-term mesocosm study. PLoS One. 2016;11: e0165800 doi: 10.1371/journal.pone.0165800 27893740 10.1371/journal.pone.0165800 PMC5125589

[51] Bach LT, Riebesell U, Gutowska MA, Federwisch L, Schulz KG. A unifying concept of coccolithophore sensitivity to changing carbonate chemistry embedded in an ecological framework. Prog Oceanogr. Elsevier Ltd; 2015;135: 125–138. doi: 10.1016/j.pocean.2015.04.012

[52] Kottmeier DM, Rokitta SD, Rost B. H^+^ -driven increase in CO_2_ uptake and decrease in HCO3- uptake explain coccolithophores’ acclimation responses to ocean acidification. Limnol Oceanogr. 2016; doi: 10.1002/lno.10352

[53] Flombaum P, Gallegos JL, Gordillo RA, Rincon J, Zabala LL, Jiao N, et al Present and future global distributions of the marine Cyanobacteria *Prochlorococcus* and *Synechococcus*. Proc Natl Acad Sci. 2013;110: 9824–9829. doi: 10.1073/pnas.1307701110 23703908 10.1073/pnas.1307701110 PMC3683724

[54] Goericke R, Repeta DJ. The pigments of *Prochlorococcus marinus*: The presence of divinylchlorophyll a and b in a marine procaryote. Limnol Oceanogr. 1992;37: 425–433. doi: 10.4319/lo.1992.37.2.0425

[55] Hunter-Cevera KR, Post AF, Peacock EE, Sosik HM. Diversity of *Synechococcus* at the Martha’s Vineyard Coastal Observatory: Insights from Culture Isolations, Clone Libraries, and Flow Cytometry. Microb Ecol. 2016;71: 276–289. doi: 10.1007/s00248-015-0644-1 26233669 10.1007/s00248-015-0644-1 PMC4728178

[56] Lomas MW, Hopkinson BM, Losh JL, Ryan DE, Shi DL, Xu Y, et al Effect of ocean acidification on cyanobacteria in the subtropical North Atlantic. Aquat Microb Ecol. 2012;66: 211–222. doi: 10.3354/ame01576

[57] McKie-Krisberg ZM, Sanders RW. Phagotrophy by the picoeukaryotic green alga *Micromonas*: implications for Arctic Oceans. ISME J. Nature Publishing Group; 2014;8: 1953–1961. doi: 10.1038/ismej.2014.16 24553471 10.1038/ismej.2014.16 PMC4184008

[58] Zubkov M V, Tarran GA. High bacterivory by the smallest phytoplankton in the North Atlantic Ocean. Nature. 2008;455: 224–226. doi: 10.1038/nature07236 18690208 10.1038/nature07236

[59] Hartmann M, Zubkov M V., Scanlan DJ, Lepère C. In situ interactions between photosynthetic picoeukaryotes and bacterioplankton in the Atlantic Ocean: evidence for mixotrophy. Environ Microbiol Rep. 2013;5: 835–840. doi: 10.1111/1758-2229.12084 24249292 10.1111/1758-2229.12084

[60] Paulino AI, Egge JK, Larsen A. Effects of increased atmospheric CO_2_ on small and intermediate sized osmotrophs during a nutrient induced phytoplankton bloom. Biogeosciences. 2008;5: 739–748.

[61] Riebesell U, Tortell PD. Effects of ocean acidification on pelagic organisms and ecosystems In: Gattuso J-P, Hansson L, editors. Ocean acidification. Oxford: Oxford University Press; 2011 pp. 99–121.

[62] Giordano M, Beardall J, Raven JA. CO_2_ concentrating mechanisms in algae: mechanisms, environmental modulation, and evolution. Annu Rev Plant Biol. 2005;56: 99–131. doi: 10.1146/annurev.arplant.56.032604.144052 15862091 10.1146/annurev.arplant.56.032604.144052

[63] Schaum E, Rost B, Millar AJ, Collins S. Variation in plastic responses of a globally distributed picoplankton species to ocean acidification. Nat Clim Chang. 2012;3: 298–302. doi: 10.1038/nclimate1774 25209938

[64] Maat DS, Crawfurd KJ, Timmermans KR, Brussaard CPD. Elevated CO_2_ and phosphate limitation favor *Micromonas pusilla* through stimulated growth and reduced viral impact. Appl Environ Microbiol. 2014;80: 3119–3127. doi: 10.1128/AEM.03639-13 24610859 10.1128/AEM.03639-13 PMC4018922

[65] Flynn KJ, Blackford JC, Baird ME, Raven JA, Clark DR, Beardall J, et al Changes in pH at the exterior surface of plankton with ocean acidification. Nat Clim Chang. Nature Publishing Group; 2012;2: 510–513. doi: 10.1038/nclimate1489

[66] Tilman D, Kilham SS, Kilham P. Phytoplankton Community Ecology: The Role of Limiting Nutrients. Annu Rev Ecol Syst. 1982;13: 349–372. doi: 10.1146/annurev.es.13.110182.002025

[67] Pasciak WJ, Gavis J. Transport limitation of nutrient uptake in phytoplankton. Limnol Oceanogr. 1974;19: 881–888.

[68] Grossart HP, Allgaier M, Passow U, Riebesell U. Testing the effect of CO_2_ concentration on the dynamics of marine heterotrophic bacterioplankton. Limnol Oceanogr. 2006;51: 1–11. doi: 10.4319/lo.2006.51.1.0001

[69] Piontek J, Borchard C, Sperling M, Schulz KG, Riebesell U, Engel A. Response of bacterioplankton activity in an Arctic fjord system to elevated pCO_2_: Results from a mesocosm perturbation study. Biogeosciences. 2013;10: 297–314. doi: 10.5194/bg-10-297-2013

[70] Endres S, Galgani L, Riebesell U, Schulz KG, Engel A. Stimulated bacterial growth under elevated pCO_2_: Results from an off-shore mesocosm study. PLoS One. 2014;9: 1–8. doi: 10.1371/journal.pone.0099228 24941307 10.1371/journal.pone.0099228 PMC4062391

[71] Maas EW, Law CS, Hall JA, Pickmere S, Currie KI, Chang FH, et al Effect of ocean acidification on bacterial abundance, activity and diversity in the Ross Sea, Antarctica. Aquat Microb Ecol. 2013;70: 1–15. doi: 10.3354/ame01633

[72] Endres S, Unger J, Wannicke N, Nausch M, Voss M, Engel A. Response of *Nodularia spumigena* to pCO2—Part 2: Exudation and extracellular enzyme activities. Biogeosciences. 2013;10: 567–582. doi: 10.5194/bg-10-567-2013

[73] Sanders RW, Gast RJ. Bacterivory by phototrophic picoplankton and nanoplankton in Arctic waters. FEMS Microbiol Ecol. 2012;82: 242–253. doi: 10.1111/j.1574-6941.2011.01253.x 22092954 10.1111/j.1574-6941.2011.01253.x

[74] Hornick T, Bach LT, Crawfurd KJ, Spilling K, Achterberg EP, Woodhouse JN, et al Ocean acidification impacts bacteria–phytoplankton coupling at low-nutrient conditions. Biogeosciences. 2017;14: 1–15. doi: 10.5194/bg-14-1-2017

[75] Thingstad TF, Havskum H, Garde K, Riemann B. On the Strategy of “Eating Your Competitor”: A Mathematical Analysis of Algal Mixotrophy. Ecology. 1996;77: 2108–2118.

[76] McKie-Krisberg ZM, Gast RJ, Sanders RW. Physiological Responses of Three Species of Antarctic Mixotrophic Phytoflagellates to Changes in Light and Dissolved Nutrients. Microb Ecol. 2015;70: 21–29. doi: 10.1007/s00248-014-0543-x 25482369 10.1007/s00248-014-0543-x

[77] Brussaard CPD, Noordeloos AAM, Witte H, Collenteur MCJ, Schulz K, Ludwig A, et al Arctic microbial community dynamics influenced by elevated CO_2_ levels. Biogeosciences. 2013;10: 719–731. doi: 10.5194/bg-10-719-2013

[78] Li S, Zhou J, Wei L, Kong F, Shi X. The effect of elevated CO_2_ on autotrophic picoplankton abundance and production in a eutrophic lake (Lake Taihu, China). Mar Freshw Res. 2016;67: 319–326. doi: 10.1071/MF14353

[79] Newbold LK, Oliver AE, Booth T, Tiwari B, Desantis T, Maguire M, et al The response of marine picoplankton to ocean acidification. Environ Microbiol. 2012;14: 2293–2307. doi: 10.1111/j.1462-2920.2012.02762.x 22591022 10.1111/j.1462-2920.2012.02762.x

[80] López-Urrutia Á, Morán XAG. Temperature affects the size-structure of phytoplankton communities in the ocean. Limnol Oceanogr. 2015;60: 733–738. doi: 10.1002/lno.10049

[81] Sommer U, Peter KH, Genitsaris S, Moustaka-Gouni M. Do marine phytoplankton follow Bergmann’s rule sensu lato? Biol Rev. 2016; doi: 10.1111/brv.12266 27028628 10.1111/brv.12266

[82] Sarmento H, Montoya JM, Vázquez-Domínguez E, Vaqué D, Gasol JM. Warming effects on marine microbial food web processes: how far can we go when it comes to predictions? Philos Trans R Soc Lond B Biol Sci. 2010;365: 2137–2149. doi: 10.1098/rstb.2010.0045 20513721 10.1098/rstb.2010.0045 PMC2880134

[83] Li WKW, McLaughlin FA, Lovejoy C, Carmack EC. Smallest algae thrive as the Arctic Ocean freshens. Science. 2009;326: 539 doi: 10.1126/science.1179798 19900890 10.1126/science.1179798

