# Supplementary material for: Simulated ocean acidification reveals winners and losers in coastal phytoplankton
Source: PLoS One. 2017 Nov 30;12(11):e0188198. doi: 10.1371/journal.pone.0188198 (PMC5708705; doi:10.1371/journal.pone.0188198)
Supplement: S1 Fig — Plots A–C and E–G show the gates for Peuks and Nano I–IV in mesocosm 4 (A = day -1, B = day 35, C = day 93) and mesocosm 10 (E = day -1, F = day 35, G = day 93). Please note that gates were adjusted in the course of the experiments to account for changing population appearances (section 2.2). Plots D and H show the gates of Synechococcus and Crypto populations, respectively. These gates remained unchanged during the entire study. (PDF) [file pone.0188198.s004.pdf]

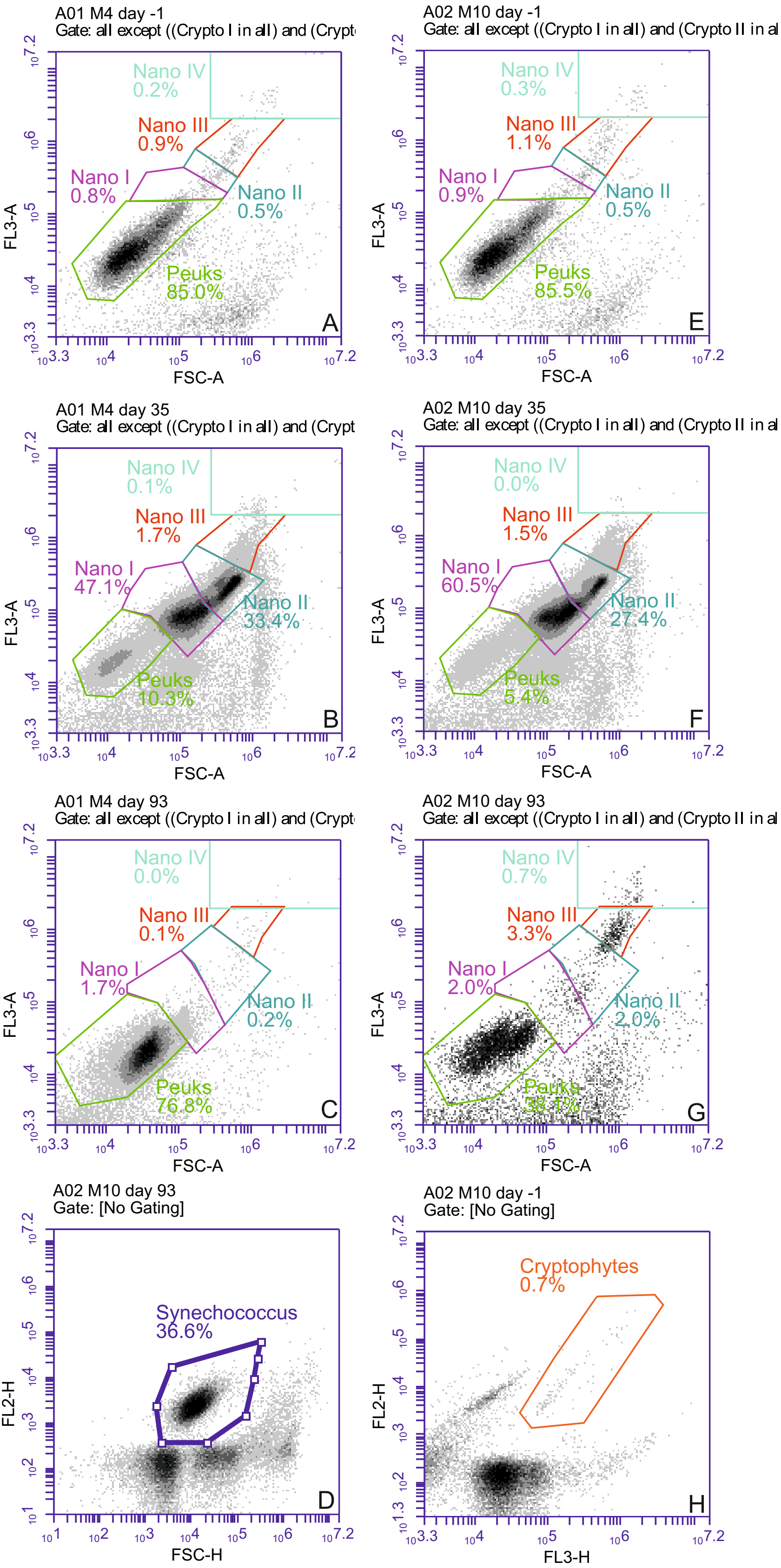

A01 M4 day -1
Gate: all except ((Crypto I in all) and (Crypt
Nano IV 0.2%
Nano III 0.9%
Nano I 0.8%
Nano II 0.5%
Peuks 85.0%
A
A02 M10 day -1
Gate: all except ((Crypto I in all) and (Crypto II in al
Nano IV 0.3%
Nano III 1.1%
Nano I 0.9%
Nano II 0.5%
Peuks 85.5%
E
A01 M4 day 35
Gate: all except ((Crypto I in all) and (Crypt
Nano IV 0.1%
Nano III 1.7%
Nano I 47.1%
Nano II 33.4%
Peuks 10.3%
B
A02 M10 day 35
Gate: all except ((Crypto I in all) and (Crypto II in al
Nano IV 0.0%
Nano III 1.5%
Nano I 60.5%
Nano II 27.4%
Peuks 5.4%
F
A01 M4 day 93
Gate: all except ((Crypto I in all) and (Crypt
Nano IV 0.0%
Nano III 0.1%
Nano I 1.7%
Nano II 0.2%
Peuks 76.8%
C
A02 M10 day 93
Gate: all except ((Crypto I in all) and (Crypto II in al
Nano IV 0.7%
Nano III 3.3%
Nano I 2.0%
Nano II 2.0%
Peuks 38.1%
G
FL3-A
FSC-A
A02 M10 day 93
Gate: [No Gating]
Synechococcus 36.6%
FL2-H
FSC-H
D
A02 M10 day -1
Gate: [No Gating]
Cryptophytes 0.7%
FL2-H
FL3-H
H
