# Supplementary figures and images for: Simulated ocean acidification reveals winners and losers in coastal phytoplankton
Source: PLoS One. 2017 Nov 30;12(11):e0188198. doi: 10.1371/journal.pone.0188198 (PMC5708705; doi:10.1371/journal.pone.0188198)

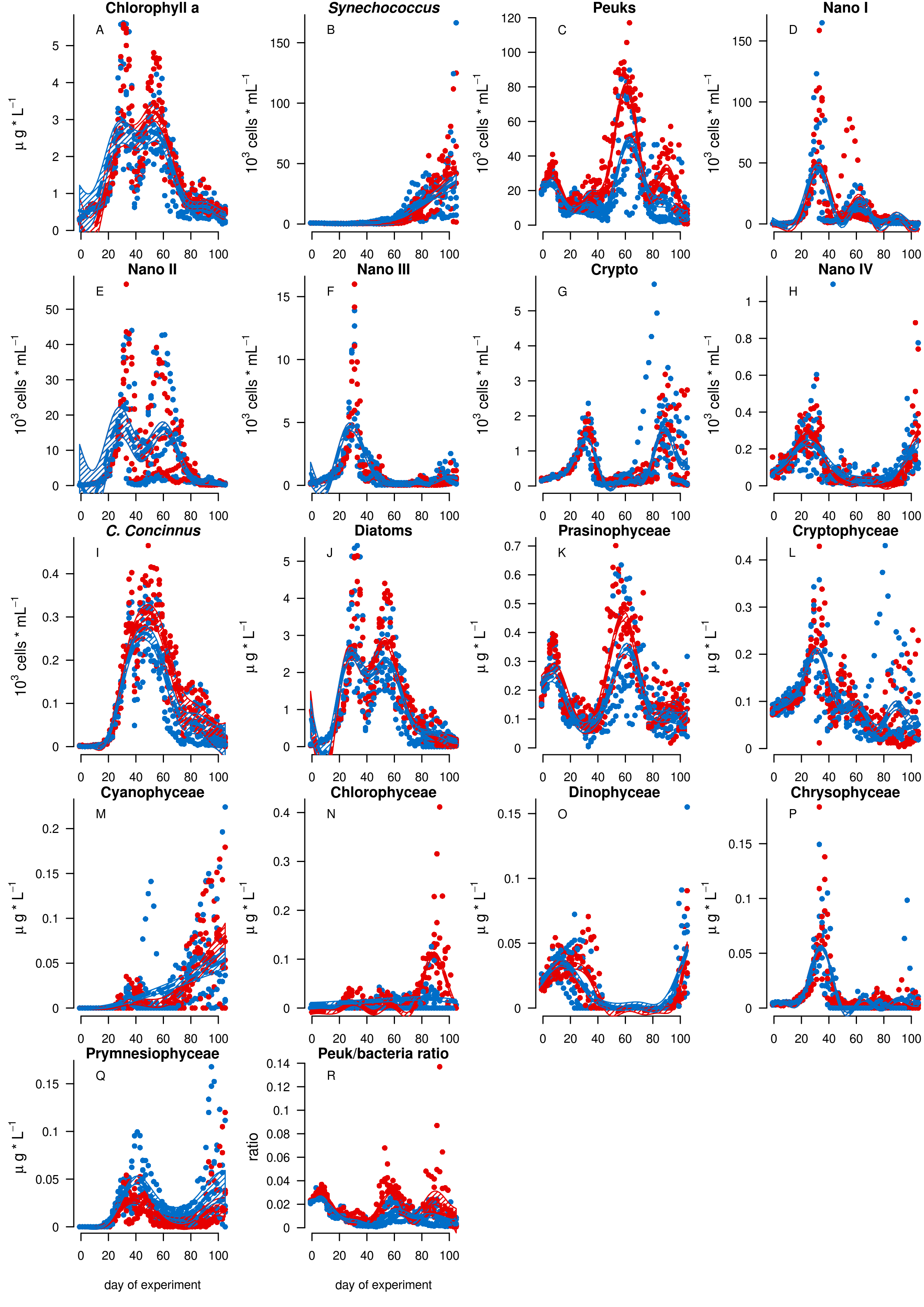

Supplement: S2 Fig — The blue and red lines are fitted GAMMs with the shaded areas representing confidence intervals. CO2 effects were detected when both a red and a blue line are present in the plots. A blue line is always present meaning that time always had a significant effect on the trends. Blue and red dots are underlying raw data from 5 control and 5 high CO2 mesocosms, respectively. A summary on the GAMM results is provided in Table 1. (TIFF) [file pone.0188198.s005.tiff]

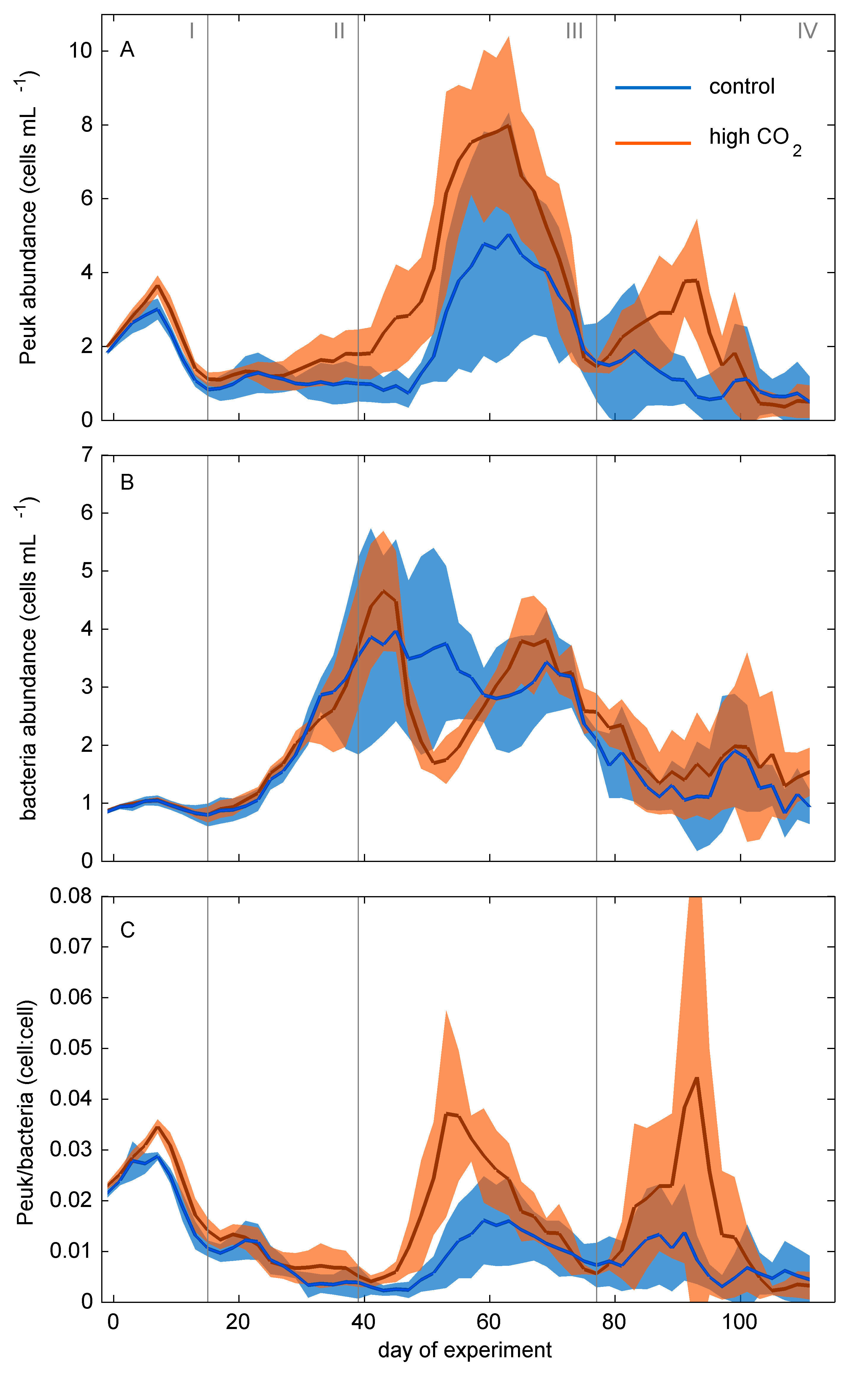

Supplement: S3 Fig — Red and blue lines show the average of five high and five ambient CO2 mesocosms, respectively. Shaded areas represent standard deviations from means. Vertical grey lines (Roman numbers I to IV) separate the four experimental phases. (A) Peuk abundance (same as in Fig 3B). (B) Bacteria abundance. (C) Peuk to bacteria abundance ratio. Statistical significance was detected in all three datasets by means of GAMM (Peuk abundance R2adj. = 0.71, bacteria abundance R2adj. = 0.72, Peuk/bacteria ratio R2adj. = 0.76). (TIF) [file pone.0188198.s006.tif]
